# Supplementary material for: Alternative splicing impacts microRNA regulation within coding regions
Source: NAR Genom Bioinform. 2023 Sep 11;5(3):lqad081. doi: 10.1093/nargab/lqad081 (PMC10495541; doi:10.1093/nargab/lqad081)
Supplement: lqad081_supplemental_file [file lqad081_supplemental_file.pdf]

SUPPLEMENTARY DATA AND FIGURES

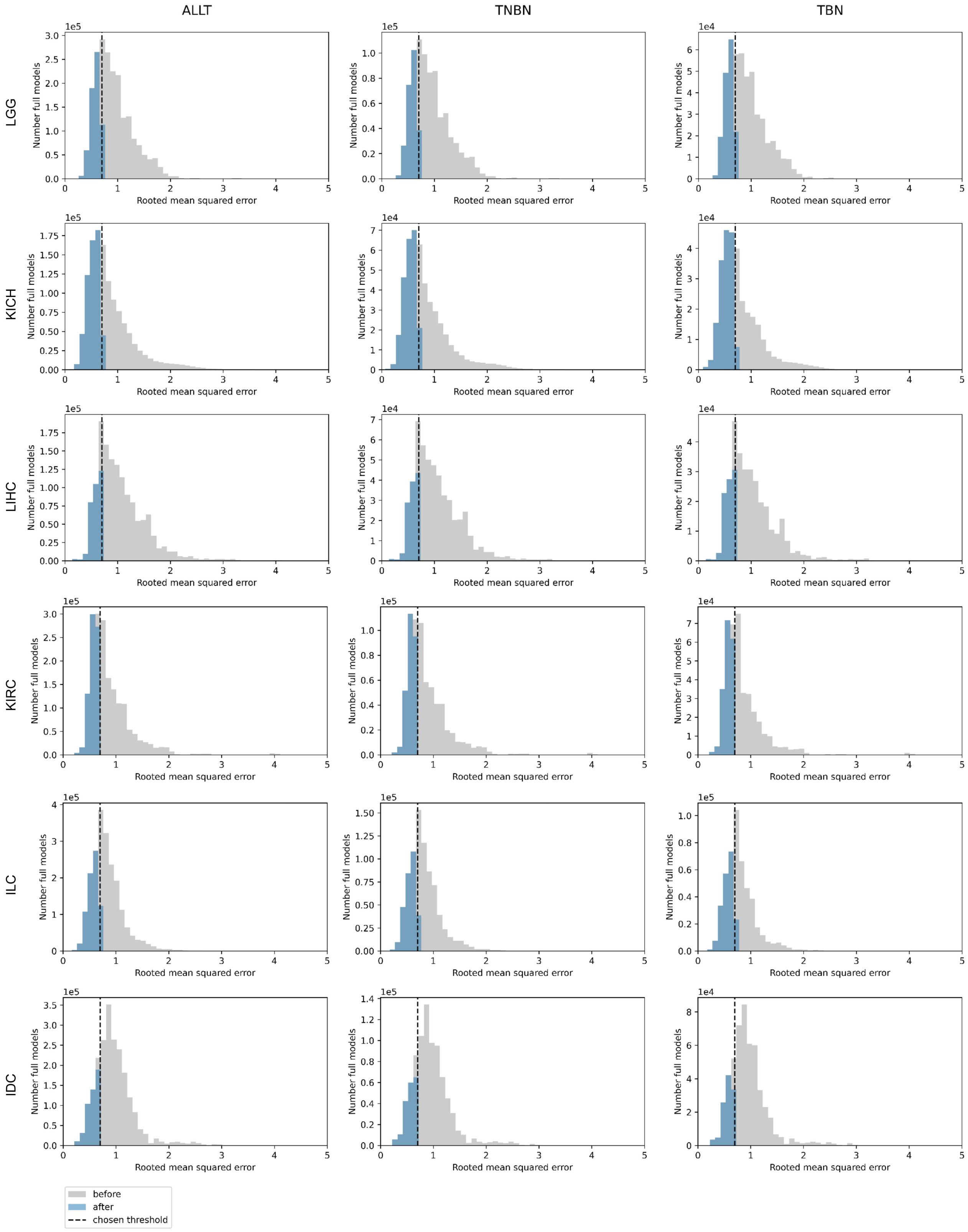

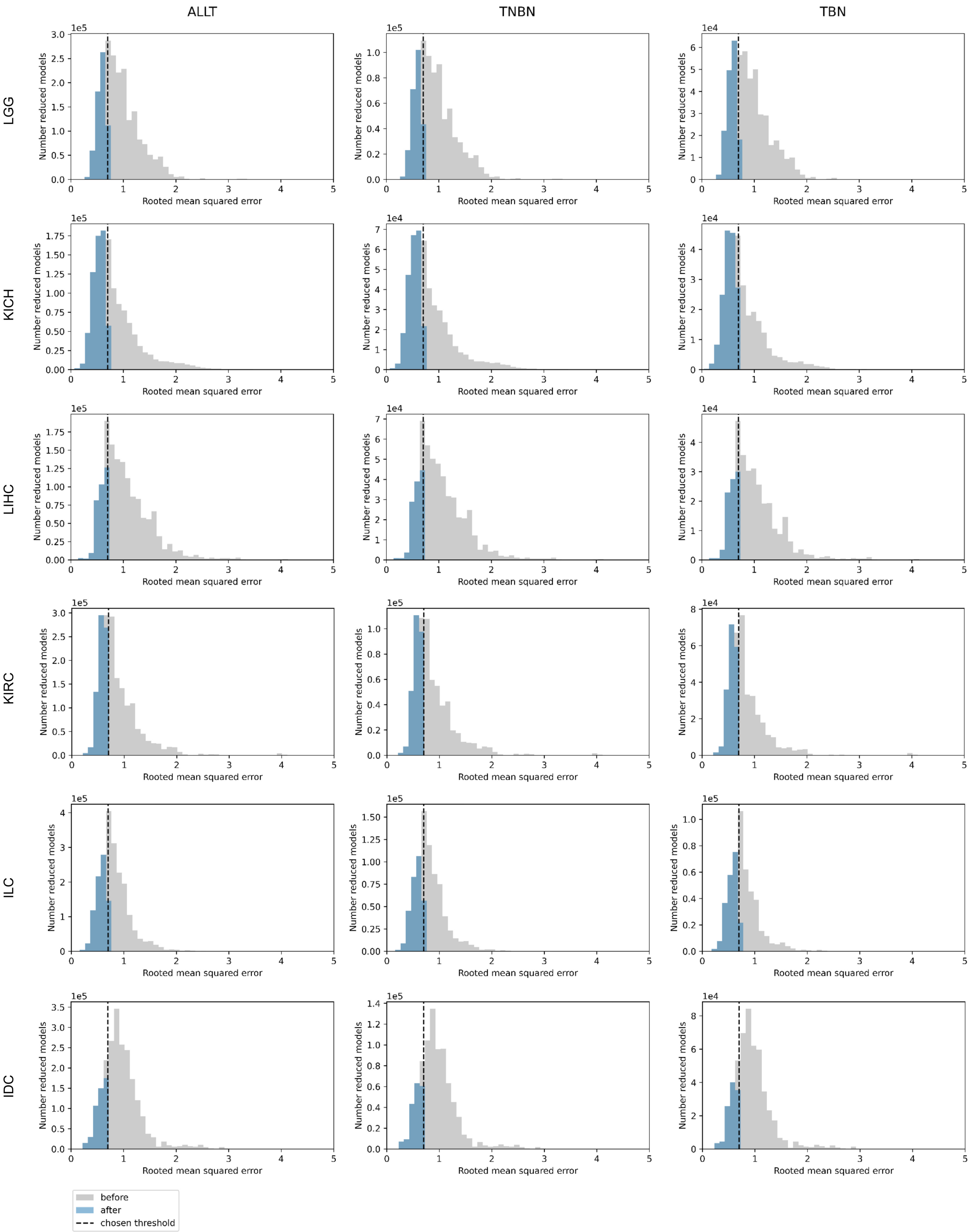

Figure S1. Amount of full and reduced models being filtered out by the rooted mean squared error filter depicted for settings ALLT, TNBN, and TBN for Brain lower grade glioma (LGG), Kidney chromophobe carcinoma (KICH), Liver hepatocellular carcinoma (LIHC), Kidney renal cell carcinoma (KIRC) and Breast Invasive Carcinoma types Invasive Lobular Carcinoma (ILC) and Invasive Ductal Carcinoma (IDC).

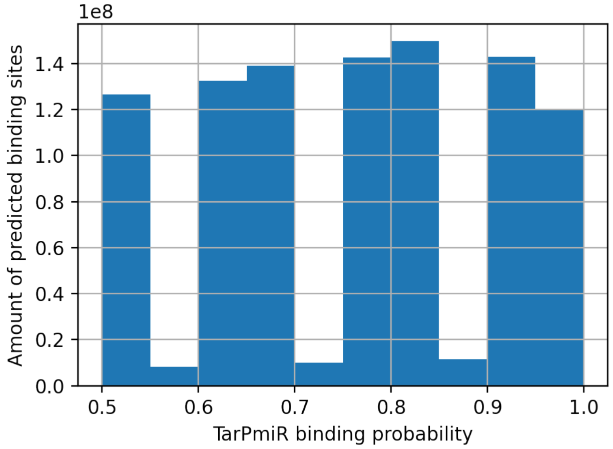

Figure S2. Binding probabilities of predicted TarPmiR target sites before any filtering

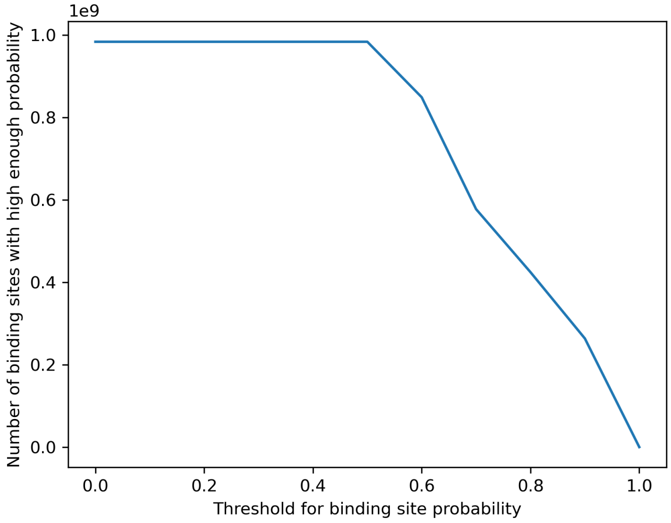

Figure S3. Amount of target sites shown for varying binding probability threshold

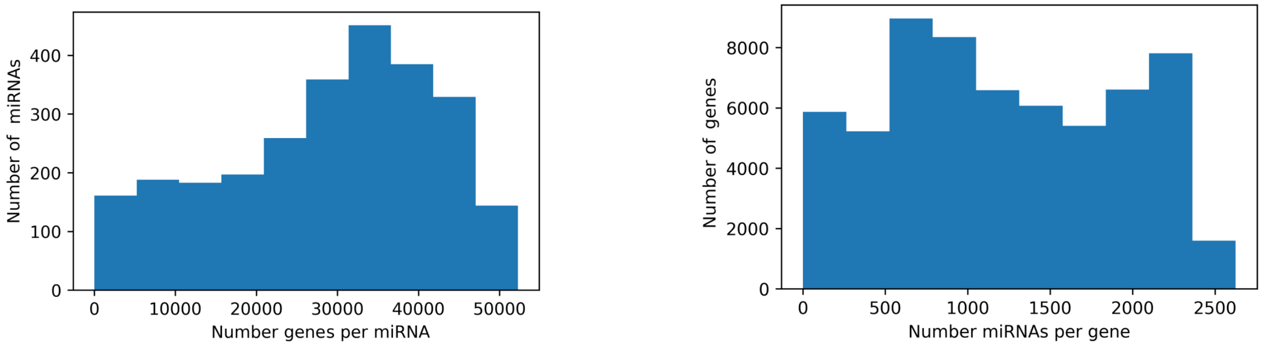

Figure S4. Amount of miRNAs per gene and genes per miRNA depicted before any filtering.

Table S1. Intermediate number of miRNA-gene pairs after different filtering steps and number final nested models shown for settings ALLT, TNBN, TBN and for the investigated cancer types.

	# of miRNA-gene pairs after expression filter			# of miRNA-gene pairs after alternative splicing filter			# of miRNA-gene models after RMSE filter		
Cancer	ALLT	TNBN	TBN	ALLT	TNBN	TBN	ALLT	TNBN	TBN
LGG	5,537,553	2,580,282	1,416,480	3,926,467	1,507,316	913,827	610,263	237,734	153,394
KICH	4,818,224	2,268,596	1,261,454	3,252,566	1,244,890	765,695	546,157	213,742	147,345
LIHC	5,970,450	2,824,880	1,557,801	3,347,063	1,217,406	759,370	313,382	114,776	83,152
KIRC	5,534,423	2,586,037	1,428,360	3,849,318	1,470,433	903,506	704,955	263,378	170,929
ILC	5,527,849	2,592,399	1,435,927	3,849,351	1,478,371	908,345	715,294	282,035	190,513
IDC	6,057,604	2,841,251	1,575,977	4,115,021	1,578,926	967,695	469,066	181,298	108,074

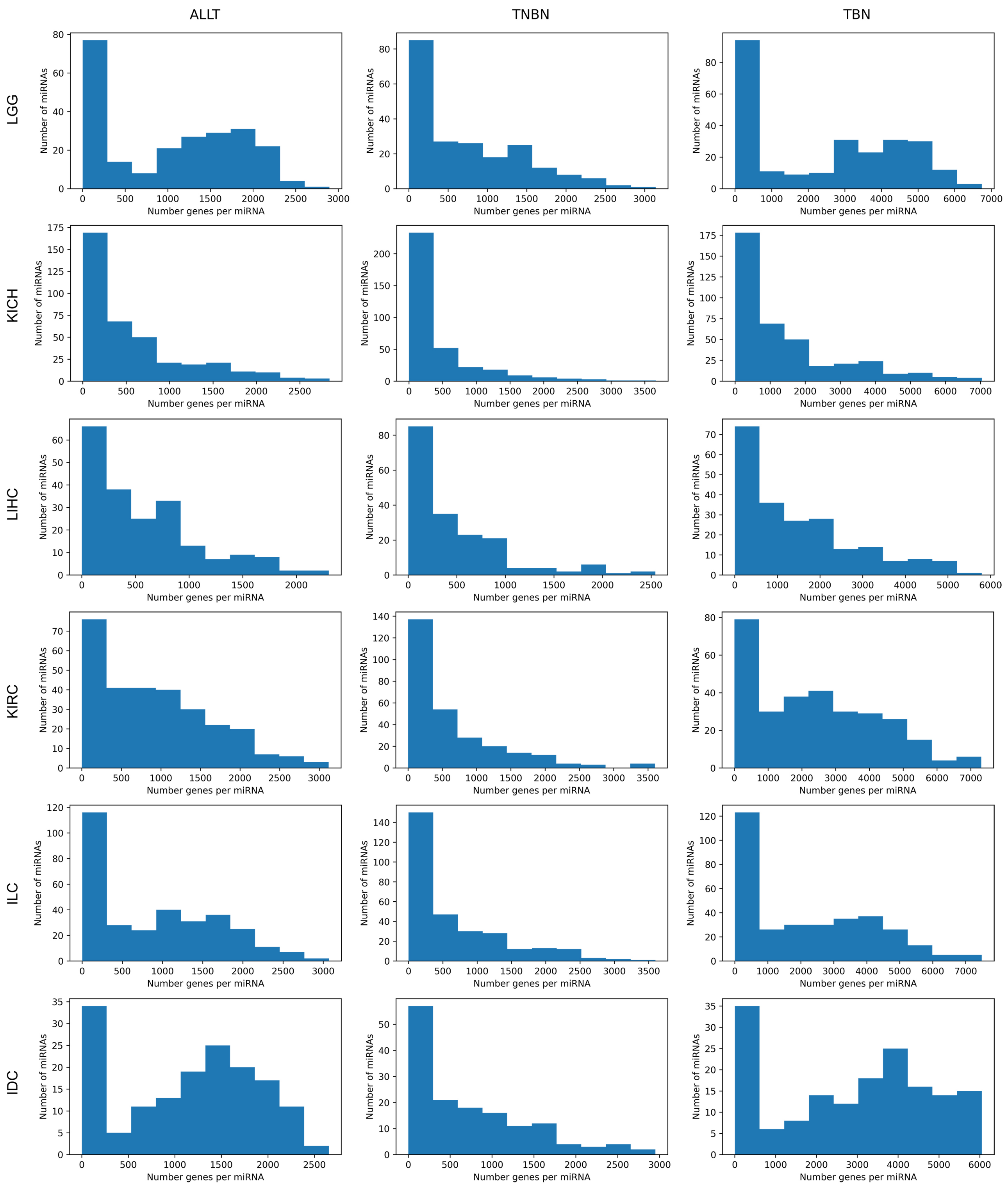
ALLT
TNBN
TBN
LGG
KICH
LIHC
KIRC
ILC
IDC
Number of miRNAs
Number genes per miRNA

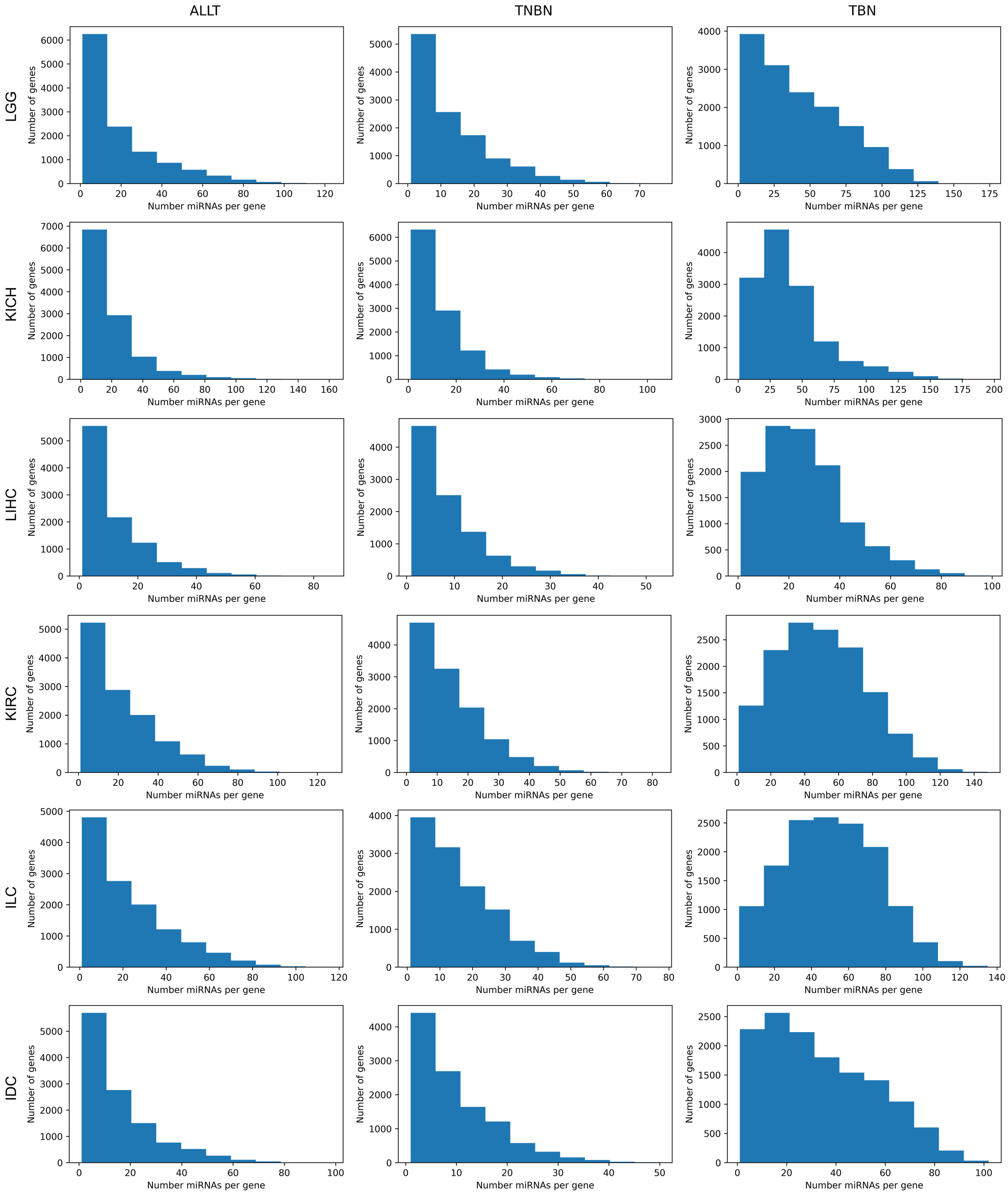

Figure S5. Amount of miRNAs per gene and genes per miRNA depicted after filtering for settings ALLT, TNBN, and TBN for Brain lower grade glioma (LGG), Kidney chromophobe carcinoma (KICH), Liver hepatocellular carcinoma (LIHC), Kidney renal cell carcinoma (KIRC) and Breast Invasive Carcinoma types Invasive Lobular Carcinoma (ILC) and Invasive Ductal Carcinoma (IDC).

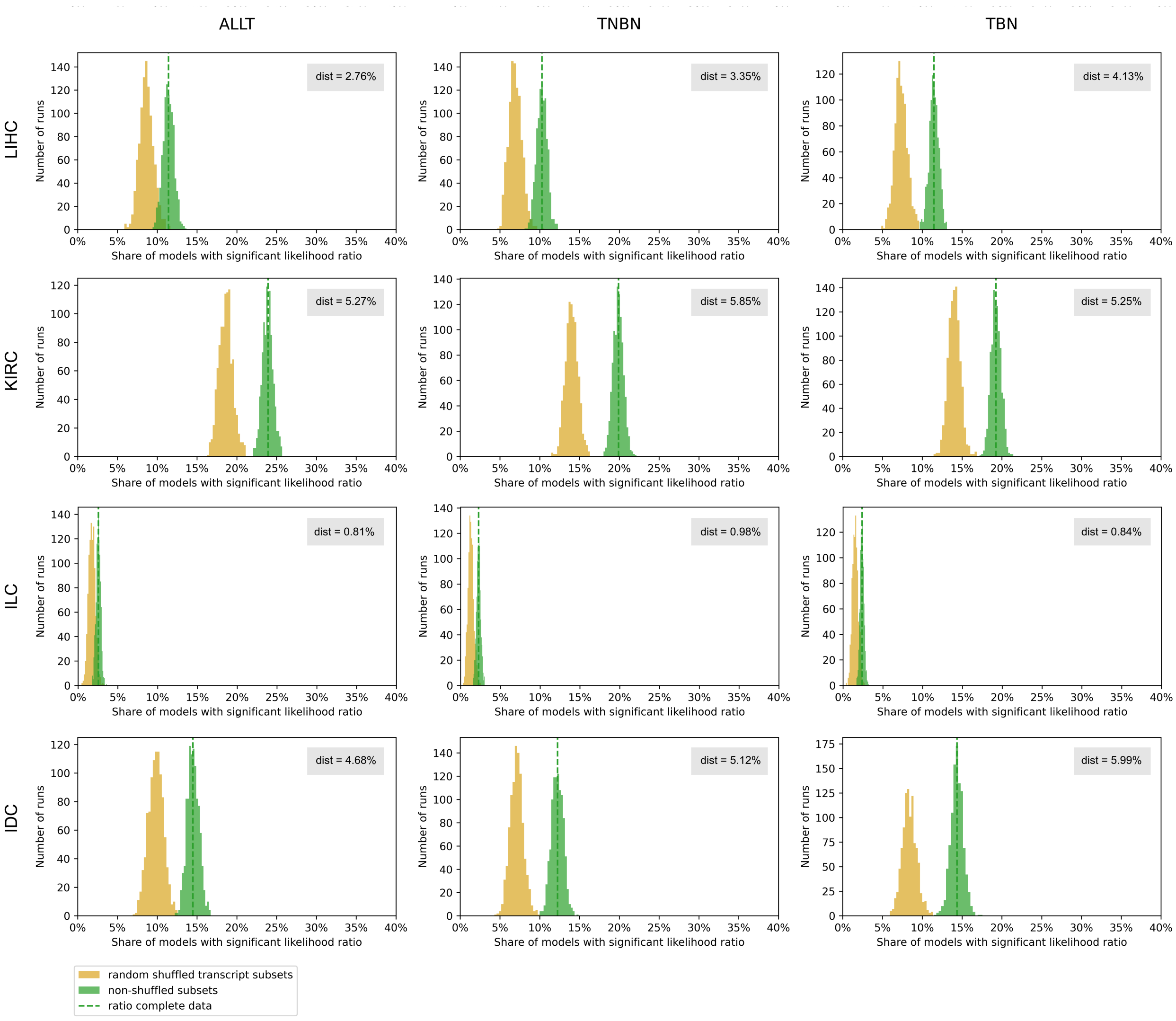

Figure S6. The ratio of models with statistically significant (<0.05) corrected p-values of the likelihood ratio test statistic calculated between nested regression models is shown as the dashed green line. To estimate the distribution, the ratio was calculated 1,000 times for random subsets of miRNA-gene pairs (green histogram) and to estimate the impact of alternative splicing, the ratio was calculated 1,000 times for random subsets of miRNA-gene pairs while randomizing the transcript binding labels within a gene (yellow histogram). Dist describes the difference between the average ratio of models based on subsampled real miRNA-gene pairs and after randomizing the transcript category labels. This is shown separately for diseases Liver hepatocellular carcinoma (LIHC), Kidney renal cell carcinoma (KIRC) and Breast Invasive Carcinoma types Invasive Lobular Carcinoma (ILC) and Invasive Ductal Carcinoma (IDC) and for settings ALLT, TNBN, and TBN.

Table S2. Top ten most significantly enriched genesets of non-computational collections (C1, C2, C3, C5, C6, C7, C8, H) for the top 500 genes of miRNA-gene pairs with most significant corrected p-values in LGG setting TNBN.

Geneset Name	# Genes in Geneset	Description	# Genes in Overlap	FDR q-value
TRAVAGLINI_LUNG_PROXIMAL_BASAL_CELL	629	-	63	2.06E-34
RUBENSTEIN_SKELETAL_MUSCLE_T_CELLS	181	-	39	3E-33
GOMF_RNA_BINDING	1968	Binding to an RNA molecule or a portion thereof. [GOC:jl, GOC:mah]	102	3.96E-33
HSIAO_HOUSEKEEPING_GENES	397	Housekeeping genes identified as expressed across 19 normal tissues.	51	4.98E-33
BUSSLINGER_DUODENAL_STEM_CELLS	311	-	46	2.17E-32
REACTOME_EUKARYOTIC_TRANSLATION_ELONGATION	94	Eukaryotic Translation Elongation	30	4.82E-31
REACTOME_METABOLISM_OF_RNA	714	Metabolism of RNA	62	4.87E-31
GOCC_SYNAPSE	1460	The junction between an axon of one neuron and a dendrite of another neuron, a muscle fiber or a glial cell. As the axon approaches the synapse it enlarges into a specialized structure, the presynaptic terminal bouton, which contains mitochondria and synaptic vesicles. At the tip of the terminal bouton is the presynaptic membrane; facing it, and separated from it by a minute cleft (the synaptic cleft) is a specialized area of membrane on the receiving cell, known as the postsynaptic membrane. In response to the arrival of nerve impulses, the presynaptic terminal bouton secretes molecules of neurotransmitters into the synaptic cleft. These diffuse across the cleft and transmit the signal to the postsynaptic membrane. [GOC:aruk, ISBN:0198506732, PMID:24619342, PMID:29383328, PMID:31998110]	85	7.97E-31
RUBENSTEIN_SKELETAL_MUSCLE_SATELLITE_CELLS	310	-	44	2.57E-30
GOBP_CYTOPLASMIC_TRANSLATION	159	The chemical reactions and pathways resulting in the formation of a protein in the cytoplasm. This is a ribosome-mediated process in which the information in messenger RNA (mRNA) is used to specify the sequence of amino acids in the protein. [GOC:hjd]	34	3.67E-29

Table S3. Top ten most significantly enriched genesets of non-computational collections (C1, C2, C3, C5, C6, C7, C8, H) for the top 500 genes of miRNA-gene pairs with most significant corrected p-values in LGG setting TBN.

Geneset Name	# Genes in Geneset	Description	# Genes in Overlap	FDR q-value
BLALOCK_ALZHEIMERS_DISEASE_UP	1671	Genes up-regulated in brain from patients with Alzheimer's disease.	95	2.58E-33
BUSSLINGER_GASTRIC_IMMUNE_CELLS	1492	-	76	4.68E-23
GRAESSMANN_APOPTOSIS_BY_DOXORUBICIN_DN	1775	Genes down-regulated in ME-A cells (breast cancer) undergoing apoptosis in response to doxorubicin [PubChem=31703].	83	4.68E-23
GOMF_RNA_BINDING	1968	Binding to an RNA molecule or a portion thereof. [GOC:jl, GOC:mah]	84	6.63E-21
BUSSLINGER_DUODENAL_IMMUNE_CELLS	911	-	57	6.63E-21
SUPT16H_TARGET_GENES	1979	Genes containing one or more binding sites for UniProt:Q9Y5B9 (SUPT16H) in their promoter regions (TSS -1000,+100 bp) as identified by GTRD version 20.06 ChIP-seq harmonization.	83	3.03E-20
SCGGAAGY_ELK1_02	1242	Genes having at least one occurrence of the highly conserved motif M3 SCGGAAGY in the regions spanning 4 kb centered on their transcription starting sites [-2kb, +2kb]. This matches the ELK1 [GeneSymbol=ELK1] transcription factor binding site V$ELK1_02 (v7.4 TRANSFAC).	65	3.56E-20
TRAVAGLINI_LUNG_PROXIMAL_BASAL_CELL	629	-	47	4.21E-20
GOCC_SYNAPSE	1460	The junction between an axon of one neuron and a dendrite of another neuron, a muscle fiber or a glial cell. As the axon approaches the synapse it enlarges into a specialized structure, the presynaptic terminal bouton, which contains mitochondria and synaptic vesicles. At the tip of the terminal bouton is the presynaptic membrane; facing it, and separated from it by a minute cleft (the synaptic cleft) is a specialized area of membrane on the receiving cell, known as the postsynaptic membrane. In response to the arrival of nerve impulses, the presynaptic terminal bouton secretes molecules of neurotransmitters into the synaptic cleft. These diffuse across the cleft and transmit the signal to the postsynaptic membrane. [GOC:aruk, ISBN:0198506732, PMID:24619342, PMID:29383328, PMID:31998110]	70	6.57E-20
MANNO_MIDBRAIN_NEUROTYPES_HOPC	366	Cell types are named using anatomical and functional mnemonics prefixed by 'm' or'h' to indicate mouse and human respectively: OMTN, oculomotor and trochlear nucleus; Sert, serotonergic; NbM, medial neuroblast; NbDA, neuroblast dopaminergic; DA0-2, dopaminergic neurons; RN, red nucleus; Gaba1-2, GABAergic neurons; mNbL1-2, lateral neuroblasts; NbML1-5, mediolateral neuroblasts; NProg, neuronal progenitor; Prog, progenitor medial floorplate (FPM), lateral floorplate (FPL), midline (M), basal plate (BP); Rgl1-3, radial glia-like cells; Mgl, microglia; Endo, endothelial cells; Peric, pericytes; Epend, ependymal; OPC, oligodendrocyte precursor cells.	37	7.48E-20

Table S4. Top ten most significantly enriched genesets of non-computational collections (C1, C2, C3, C5, C6, C7, C8, H) for the top 500 genes of miRNA-gene pairs with most significant corrected p-values in LGG setting ALLT.

Geneset Name	# Genes in Geneset	Description	# Genes in Overlap	FDR q-value
RUBENSTEIN_SKELETAL_MUSCLE_SATELLITE_CELLS	310	-	46	7.13E-32
TRAVAGLINI_LUNG_PROXIMAL_BASAL_CELL	629	-	58	7.83E-30
GOCC_SYNAPSE	1460	The junction between an axon of one neuron and a dendrite of another neuron, a muscle fiber or a glial cell. As the axon approaches the synapse it enlarges into a specialized structure, the presynaptic terminal bouton, which contains mitochondria and synaptic vesicles. At the tip of the terminal bouton is the presynaptic membrane; facing it, and separated from it by a minute cleft (the synaptic cleft) is a specialized area of membrane on the receiving cell, known as the postsynaptic membrane. In response to the arrival of nerve impulses, the presynaptic terminal bouton secretes molecules of neurotransmitters into the synaptic cleft. These diffuse across the cleft and transmit the signal to the postsynaptic membrane. [GOC:aruk, ISBN:0198506732, PMID:24619342, PMID:29383328, PMID:31998110]	82	2.9E-28
GRAESSMANN_APOPTOSIS_BY_DOXORUBICIN_DN	1775	Genes down-regulated in ME-A cells (breast cancer) undergoing apoptosis in response to doxorubicin [PubChem=31703].	88	8.49E-27
BUSSLINGER_DUODENAL_STEM_CELLS	311	-	41	1.31E-26
RUBENSTEIN_SKELETAL_MUSCLE_T_CELLS	181	-	33	1.1E-25
REACTOME_INFECTIOUS_DISEASE	1019	Infectious disease	62	1.24E-22
BUSSLINGER_DUODENAL_DIFFERENTIATING_STEM_CELLS	305	-	37	1.24E-22
SETD1A_TARGET_GENES	1531	Genes containing one or more binding sites for UniProt:O15047 (SETD1A) in their promoter regions (TSS -1000,+100 bp) as identified by GTRD version 20.06 ChIP-seq harmonization.	75	2.97E-22
BLALOCK_ALZHEIMERS_DISEASE_UP	1671	Genes up-regulated in brain from patients with Alzheimer's disease.	78	5.03E-22

Table S5. Top ten most significantly enriched genesets of non-computational collections (C1, C2, C3, C5, C6, C7, C8, H) for the top 500 genes of miRNA-gene pairs with most significant corrected p-values in LIHC setting TNBN.

Geneset Name	# Genes in Geneset	Description	# Genes in Overlap	FDR q-value
HSIAO_LIVER_SPECIFIC_GENES	249	Liver selective genes	50	1.21E-41
GOMF_RNA_BINDING	1968	Binding to an RNA molecule or a portion thereof. [GOC:jl, GOC:mah]	108	1.63E-37
PUJANA_BRCA1_PCC_NETWORK	1625	Genes constituting the BRCA1-PCC network of transcripts whose expression positively correlated (Pearson correlation coefficient, PCC $>=$ 0.4) with that of BRCA1 [GeneID=672] across a compendium of normal tissues.	97	2.62E-36
AIZARANI_LIVER_C11_HEPATOCYTES_1	298	-	48	1.23E-35
AIZARANI_LIVER_C14_HEPATOCYTES_2	226	-	43	6.33E-35
REACTOME_METABOLISM_OF_RNA	714	Metabolism of RNA	63	6.22E-32
CAIRO_HEPATOBLASTOMA_CLASSES_UP	612	Genes up-regulated in robust Cluster 2 (rC2) of hepatoblastoma samples compared to those in the robust Cluster 1 (rC1).	59	7.01E-32
GRAESSMANN_APOPTOSIS_BY_DOXORUBICIN_DN	1775	Genes down-regulated in ME-A cells (breast cancer) undergoing apoptosis in response to doxorubicin [PubChem=31703].	90	2.47E-28
GOCC_NUCLEAR_PROTEIN_CONTAINING_COMPLEX	1243	A stable assembly of two or more macromolecules, i.e. proteins, nucleic acids, carbohydrates or lipids, in which at least one component is a protein and the constituent parts function together in the nucleus. [GOC:pg]	73	3.27E-26
TRAVAGLINI_LUNG_PROLIFERATING_BASAL_CELL	891	-	62	8.72E-26

Table S6. Top ten most significantly enriched genesets of non-computational collections (C1, C2, C3, C5, C6, C7, C8, H) for the top 500 genes of miRNA-gene pairs with most significant corrected p-values in LIHC setting TBN.

Geneset Name	# Genes in Geneset	Description	# Genes in Overlap	FDR q-value
CARRILLOREIXACH_HEPATOBLASTOMA_VS_NORMAL_DN	1259	Genes down-regulated in hepatoblastoma (HB) tumors as compared with non-tumor (NT) adjacent tissue.	105	4E-53
GOBP_SMALL_MOLECULE_METABOLIC_PROCESS	1830	The chemical reactions and pathways involving small molecules, any low molecular weight, monomeric, non-encoded molecule. [GOC:curators, GOC:pde, GOC:vw]	117	4.6E-48
HOSHIDA_LIVER_CANCER_SUBCLASS_S3	266	Genes from 'subtype S3' signature of hepatocellular carcinoma (HCC): hepatocyte differentiation.	52	2.4E-43
HSIAO_LIVER_SPECIFIC_GENES	249	Liver selective genes	49	6.38E-41
AIZARANI_LIVER_C11_HEPATOCYTES_1	298	-	51	1.38E-39
GOBP_ORGANIC_ACID_METABOLIC_PROCESS	969	The chemical reactions and pathways involving organic acids, any acidic compound containing carbon in covalent linkage. [ISBN:0198506732]	77	1.06E-36
AIZARANI_LIVER_C14_HEPATOCYTES_2	226	-	42	8.88E-34
CHIANG_LIVER_CANCER_SUBCLASS_PROLIFERATION_DN	179	Top 200 marker genes down-regulated in the 'proliferation' subclass of hepatocellular carcinoma (HCC); characterized by increased proliferation, high levels of serum AFP [GeneID=174], and chromosomal instability.	36	6.23E-30
GOCC_MITOCHONDRION	1672	A semiautonomous, self replicating organelle that occurs in varying numbers, shapes, and sizes in the cytoplasm of virtually all eukaryotic cells. It is notably the site of tissue respiration. [GOC:giardia, ISBN:0198506732]	87	3.55E-28
FLECHNER_BIOPSY_KIDNEY_TRANSPLANT_REJECTED_VS_OK_DN	553	Genes down-regulated in kidney biopsies from patients with acute transplant rejection compared to the biopsies from patients with well functioning kidneys more than 1-year post transplant.	52	2.12E-27

Table S7. Top ten most significantly enriched genesets of non-computational collections (C1, C2, C3, C5, C6, C7, C8, H) for the top 500 genes of miRNA-gene pairs with most significant corrected p-values in LIHC setting ALLT.

Geneset Name	# Genes in Geneset	Description	# Genes in Overlap	FDR q-value
GOMF_RNA_BINDING	1968	Binding to an RNA molecule or a portion thereof. [GOC:jl, GOC:mah]	115	2.98E-43
PUJANA_BRCA1_PCC_NETWORK	1625	Genes constituting the BRCA1-PCC network of transcripts whose expression positively correlated (Pearson correlation coefficient, PCC $>=$ 0.4) with that of BRCA1 [GeneID=672] across a compendium of normal tissues.	103	1.68E-41
CAIRO_HEPATOBLASTOMA_CLASSES_UP	612	Genes up-regulated in robust Cluster 2 (rC2) of hepatoblastoma samples compared to those in the robust Cluster 1 (rC1).	66	4.82E-39
REACTOME_METABOLISM_OF_RNA	714	Metabolism of RNA	66	5.76E-35
DODD_NASOPHARYNGEAL_CARCINOMA_DN	1405	Genes down-regulated in nasopharyngeal carcinoma (NPC) compared to the normal tissue.	87	8.25E-34
ELF2_TARGET_GENES	1511	Genes containing one or more binding sites for UniProt:Q15723 (ELF2) in their promoter regions (TSS -1000,+100 bp) as identified by GTRD version 20.06 ChIP-seq harmonization.	87	1.65E-31
HE_LIM_SUN_FETAL_LUNG_C5_LARGE_PRE_B_CELL	1363	Large pre-B	80	2.87E-29
HOUNKPE_HOUSEKEEPING_GENES	1129	List of 1130 human and mouse housekeeping genes. This list shows the overlap of human genes stably expressed across 52 tissues and cells types and mouse genes with at least one of their transcripts expressed across 14 tissues and cells types.	71	1.91E-27
GOCC_NUCLEAR_PROTEIN_CONTAINING_COMPLEX	1243	A stable assembly of two or more macromolecules, i.e. proteins, nucleic acids, carbohydrates or lipids, in which at least one component is a protein and the constituent parts function together in the nucleus. [GOC:pg]	73	1.57E-26
FISCHER_DREAM_TARGETS	969	Target genes of the DREAM complex.	65	1.57E-26

Table S8. Top ten most significantly enriched genesets of non-computational collections (C1, C2, C3, C5, C6, C7, C8, H) for the top 500 genes of miRNA-gene pairs with most significant corrected p-values in KIRC setting TNBN.

Geneset Name	# Genes in Geneset	Description	# Genes in Overlap	FDR q-value
MURARO_PANCREAS_DUCTAL_CELL	1276	-	82	8.39E-32
BUSSLINGER_GASTRIC_IMMUNE_CELLS	1492	-	75	3.54E-22
HP_ABNORMAL_CIRCULATING_METABOLITE_CONCENTRATION	1283	Abnormal circulating metabolite concentration	68	3.23E-21
GOBP_CELL_ADHESION	1524	The attachment of a cell, either to another cell or to an underlying substrate such as the extracellular matrix, via cell adhesion molecules. [GOC:hb, GOC:pf]	74	3.23E-21
GOBP_CYTOSKELETON_ORGANIZATION	1509	A process that is carried out at the cellular level which results in the assembly, arrangement of constituent parts, or disassembly of cytoskeletal structures. [GOC:dph, GOC:jl, GOC:mah]	71	1.62E-19
BLALOCK_ALZHEIMERS_DISEASE_UP	1671	Genes up-regulated in brain from patients with Alzheimer's disease.	74	5.1E-19
GRAESSMANN_APOPTOSIS_BY_DOXORUBICIN_DN	1775	Genes down-regulated in ME-A cells (breast cancer) undergoing apoptosis in response to doxorubicin [PubChem=31703].	75	3.43E-18
LAKE_ADULT_KIDNEY_C3_PROXIMAL_TUBULE_EPITHELIAL_CELLS_S1_S2	221	-	29	3.8E-18
GOBP_SMALL_MOLECULE_METABOLIC_PROCESS	1830	The chemical reactions and pathways involving small molecules, any low molecular weight, monomeric, non-encoded molecule. [GOC:curators, GOC:pde, GOC:vw]	76	3.8E-18
NAKAYA_PBMC_FLUMIST_AGE_18_50YO_7DY_UP	1575	Genes up-regulated in peripheral blood mononuclear cell 7d vs 0d in adults (18-50) after exposure to FluMist , time point 7D. Comment: Supplementary Table 1b: All the differentially expressed genes identified in PBMCs of TIV vaccinees.	70	4.12E-18

Table S9. Top ten most significantly enriched genesets of non-computational collections (C1, C2, C3, C5, C6, C7, C8, H) for the top 500 genes of miRNA-gene pairs with most significant corrected p-values in KIRC setting TBN.

Geneset Name	# Genes in Geneset	Description	# Genes in Overlap	FDR q-value
MURARO_PANCREAS_DUCTAL_CELL	1276	-	77	9.81E-28
BUSSLINGER_GASTRIC_IMMUNE_CELLS	1492	-	79	4.56E-25
GOBERT_OLIGODENDROCYTE_DIFFERENTIATION_DN	1088	Genes down-regulated during differentiation of Oli-Neu cells (oligodendroglial precursor) in response to PD174265 [PubChem=4709].	64	3.52E-22
NAKAYA_PBMC_FLUMIST_AGE_18_50YO_7DY_UP	1575	Genes up-regulated in peripheral blood mononuclear cell 7d vs 0d in adults (18-50) after exposure to FluMist , time point 7D. Comment: Supplementary Table 1b: All the differentially expressed genes identified in PBMCs of TIV vaccinees.	71	2.3E-18
DODD_NASOPHARYNGEAL_CARCINOMA_UP	1813	Genes up-regulated in nasopharyngeal carcinoma (NPC) compared to the normal tissue.	76	3.43E-18
GOCC_ANCHORING_JUNCTION	899	A cell junction that mechanically attaches a cell (and its cytoskeleton) to neighboring cells or to the extracellular matrix. [ISBN:0815332181]	53	3.43E-18
GRAESSMANN_APOPTOSIS_BY_DOXORUBICIN_DN	1775	Genes down-regulated in ME-A cells (breast cancer) undergoing apoptosis in response to doxorubicin [PubChem=31703].	75	3.43E-18
MIR8485	1034	Genes predicted to be targets of miRBase v22 microRNA hsa-miR-8485 in miRDB v6.0 with MirTarget v4 prediction scores $>$ 80 (high confidence targets).	56	1.05E-17
GOBP_SMALL_MOLECULE_METABOLIC_PROCESS	1830	The chemical reactions and pathways involving small molecules, any low molecular weight, monomeric, non-encoded molecule. [GOC:curators, GOC:pde, GOC:vw]	74	6.38E-17
MANNO_MIDBRAIN_NEUROTYPES_HENDO	888	Cell types are named using anatomical and functional mnemonics prefixed by 'm' or'h' to indicate mouse and human respectively: OMTN, oculomotor and trochlear nucleus; Sert, serotonergic; NbM, medial neuroblast; NbDA, neuroblast dopaminergic; DA0-2, dopaminergic neurons; RN, red nucleus; Gaba1-2, GABAergic neurons; mNbL1-2, lateral neuroblasts; NbML1-5, mediolateral neuroblasts; NProg, neuronal progenitor; Prog, progenitor medial floorplate (FPM), lateral floorplate (FPL), midline (M), basal plate (BP); Rgl1-3, radial glia-like cells; Mgl, microglia; Endo, endothelial cells; Peric, pericytes; Epend, ependymal; OPC, oligodendrocyte precursor cells.	50	2.59E-16

Table S10. Top ten most significantly enriched genesets of non-computational collections (C1, C2, C3, C5, C6, C7, C8, H) for the top 500 genes of miRNA-gene pairs with most significant corrected p-values in KIRC setting ALLT.

Geneset Name	# Genes in Geneset	Description	# Genes in Overlap	FDR q-value
MURARO_PANCREAS_DUCTAL_CELL	1276	-	80	3.22E-30
KRIEG_HYPOXIA_NOT_VIA_KDM3A	746	Genes induced under hypoxia independently of KDM3A [GeneID=55818] in RCC4 cells (renal carcinoma) expressing VHL [GeneID=7428].	55	3.7E-23
BUSSLINGER_GASTRIC_IMMUNE_CELLS	1492	-	70	5.96E-19
GOBP_REGULATION_OF_INTRACELLULAR_SIGNAL_TRANSDUCTION	1770	Any process that modulates the frequency, rate or extent of intracellular signal transduction. [GOC:dph, GOC:signaling, GOC:tb, GOC:TermGenie]	74	1.49E-17
TRAVAGLINI_LUNG_PROXIMAL_CILIATED_CELL	1770	-	74	1.49E-17
GRAESSMANN_APOPTOSIS_BY_DOXORUBICIN_DN	1775	Genes down-regulated in ME-A cells (breast cancer) undergoing apoptosis in response to doxorubicin [PubChem=31703].	74	1.49E-17
ONKEN_UVEAL_MELANOMA_UP	790	Genes up-regulated in uveal melanoma: class 2 vs class 1 tumors.	48	8.76E-17
GOBP_SMALL_MOLECULE_METABOLIC_PROCESS	1830	The chemical reactions and pathways involving small molecules, any low molecular weight, monomeric, non-encoded molecule. [GOC:curators, GOC:pde, GOC:vw]	73	2.55E-16
ZNF768_TARGET_GENES	1362	Genes containing one or more binding sites for UniProt:Q9H5H4 (ZNF768) in their promoter regions (TSS -1000,+100 bp) as identified by GTRD version 20.06 ChIP-seq harmonization.	62	3.33E-16
GRYDER_PAX3FOXO1_ENHANCERS_IN_TADS	1009	Expressed genes (FPKM$>$1) associated with high-confidence PAX3-FOXO1 sites with enhancers in primary tumors and cell lines, restricted to those within topological domain boundaries	53	3.71E-16

Table S11. Top ten most significantly enriched genesets of non-computational collections (C1, C2, C3, C5, C6, C7, C8, H) for the top 500 genes of miRNA-gene pairs with most significant corrected p-values in KICH setting TNBN.

Geneset Name	# Genes in Geneset	Description	# Genes in Overlap	FDR q-value
CHARAFE_BREAST_CANCER_LUMINAL_VS_MESENCHYMAL_UP	454	Genes up-regulated in luminal-like breast cancer cell lines compared to the mesenchymal-like ones.	52	1.08E-30
TRAVAGLINI_LUNG_PROXIMAL_CILIATED_CELL	1770	-	87	8.31E-26
GOCC_MITOCHONDRION	1672	A semiautonomous, self replicating organelle that occurs in varying numbers, shapes, and sizes in the cytoplasm of virtually all eukaryotic cells. It is notably the site of tissue respiration. [GOC:giardia, ISBN:0198506732]	82	3.62E-24
DODD_NASOPHARYNGEAL_CARCINOMA_UP	1813	Genes up-regulated in nasopharyngeal carcinoma (NPC) compared to the normal tissue.	84	2.66E-23
MURARO_PANCREAS_DUCTAL_CELL	1276	-	69	2.09E-22
BLALOCK_ALZHEIMERS_DISEASE_UP	1671	Genes up-regulated in brain from patients with Alzheimer's disease.	79	2.09E-22
CHARAFE_BREAST_CANCER_LUMINAL_VS_BASAL_UP	384	Genes up-regulated in luminal-like breast cancer cell lines compared to the basal-like ones.	40	4.96E-22
LIM_MAMMARY_STEM_CELL_DN	416	Genes consistently down-regulated in mammary stem cells both in mouse and human species.	41	9.02E-22
GOBP_CYTOSKELETON_ORGANIZATION	1509	A process that is carried out at the cellular level which results in the assembly, arrangement of constituent parts, or disassembly of cytoskeletal structures. [GOC:dph, GOC:jl, GOC:mah]	71	8E-20
STEIN_ESRRA_TARGETS_UP	385	Genes up-regulated by ESRRA [GeneID=2101] only.	37	4.08E-19

Table S12. Top ten most significantly enriched genesets of non-computational collections (C1, C2, C3, C5, C6, C7, C8, H) for the top 500 genes of miRNA-gene pairs with most significant corrected p-values in KICH setting TBN.

Geneset Name	# Genes in Geneset	Description	# Genes in Overlap	FDR q-value
RODRIGUES_THYROID_CARCINOMA_POORLY_DIFFERENTIATED_DN	802	Genes down-regulated in poorly differentiated thyroid carcinoma (PDTC) compared to normal thyroid tissue.	61	1.98E-26
LAKE_ADULT_KIDNEY_C20_COLLECTING_DUCT_INTERCALATED_CELLS_TYPE_A_CORTEX	152	-	32	2.24E-26
CHARAFE_BREAST_CANCER_LUMINAL_VS_MESENCHYMAL_UP	454	Genes up-regulated in luminal-like breast cancer cell lines compared to the mesenchymal-like ones.	44	9.35E-23
GOBP_SMALL_MOLECULE_METABOLIC_PROCESS	1830	The chemical reactions and pathways involving small molecules, any low molecular weight, monomeric, non-encoded molecule. [GOC:curators, GOC:pde, GOC:vw]	81	6.42E-21
STEIN_ESRRA_TARGETS	529	Genes regulated by ESRRA [GeneID=2101] in MCF-7 cells (breast cancer).	44	2.89E-20
LIM_MAMMARY_STEM_CELL_DN	416	Genes consistently down-regulated in mammary stem cells both in mouse and human species.	39	1.33E-19
STEIN_ESRRA_TARGETS_UP	385	Genes up-regulated by ESRRA [GeneID=2101] only.	37	6.69E-19
MURARO_PANCREAS_DUCTAL_CELL	1276	-	64	7.48E-19
TGACCTY_ERR1_Q2	1064	Genes having at least one occurrence of the highly conserved motif M25 TGACCTY in the regions spanning 4 kb centered on their transcription starting sites [-2kb, +2kb]. This matches the ESRRA [GeneSymbol=ESRRA] transcription factor binding site V$ERR1_Q2 (v7.4 TRANSFAC).	58	1.38E-18
LAKE_ADULT_KIDNEY_C21_COLLECTING_DUCT_INTERCALATED_CELLS_TYPE_B	103	-	22	4.65E-18

Table S13. Top ten most significantly enriched genesets of non-computational collections (C1, C2, C3, C5, C6, C7, C8, H) for the top 500 genes of miRNA-gene pairs with most significant corrected p-values in KICH setting ALLT.

Geneset Name	# Genes in Geneset	Description	# Genes in Overlap	FDR q-value
CHARAFE_BREAST_CANCER_LUMINAL_VS_MESENCHYMAL_UP	454	Genes up-regulated in luminal-like breast cancer cell lines compared to the mesenchymal-like ones.	47	2.14E-25
RODRIGUES_THYROID_CARCINOMA_POORLY_DIFFERENTIATED_DN	802	Genes down-regulated in poorly differentiated thyroid carcinoma (PDTC) compared to normal thyroid tissue.	58	3.49E-24
LAKE_ADULT_KIDNEY_C12_THICK_ASCENDING_LIMB	381	-	41	7.82E-23
CHEMNITZ_RESPONSE_TO_PROSTAGLANDIN_E2_DN	408	Genes down-regulated in CD4+ [GeneID=920] T lymphocytes after stimulation with prostaglandin E2 [PubChem=5280360].	39	8.52E-20
LIM_MAMMARY_STEM_CELL_DN	416	Genes consistently down-regulated in mammary stem cells both in mouse and human species.	39	1.39E-19
MURARO_PANCREAS_DUCTAL_CELL	1276	-	64	8.03E-19
TRAVAGLINI_LUNG_PROXIMAL_CILIATED_CELL	1770	-	75	2.57E-18
DODD_NASOPHARYNGEAL_CARCINOMA_UP	1813	Genes up-regulated in nasopharyngeal carcinoma (NPC) compared to the normal tissue.	74	3.75E-17
MENON_FETAL_KIDNEY_8_CONNECTING_TUBULE_CELLS	267	-	30	5.96E-17
COLDREN_GEFITINIB_RESISTANCE_DN	229	Genes down-regulated in NSCLC (non-small cell lung carcinoma) cell lines resistant to gefitinib [PubChem=123631] compared to the sensitive ones.	28	1.04E-16

Table S14. Top ten most significantly enriched genesets of non-computational collections (C1, C2, C3, C5, C6, C7, C8, H) for the top 500 genes of miRNA-gene pairs with most significant corrected p-values in ILC setting TNBN.

Geneset Name	# Genes in Geneset	Description	# Genes in Overlap	FDR q-value
DIAZ_CHRONIC_MYELOGENOUS_LEUKEMIA_UP	1399	Genes up-regulated in CD34+ [GeneID=947] cells isolated from bone marrow of CML (chronic myelogenous leukemia) patients, compared to those from normal donors.	79	1.45E-26
PUJANA_BRCA1_PCC_NETWORK	1625	Genes constituting the BRCA1-PCC network of transcripts whose expression positively correlated (Pearson correlation coefficient, PCC >= 0.4) with that of BRCA1 [GeneID=672] across a compendium of normal tissues.	79	1.37E-22
BLALOCK_ALZHEIMERS_DISEASE_UP	1671	Genes up-regulated in brain from patients with Alzheimer's disease.	79	5.48E-22
HP_PEDIATRIC_ONSET	1592	Pediatric onset	76	2.21E-21
HP_ABNORMALITY_OF_THE_FOREHEAD	1128	Abnormality of the forehead	63	9.53E-21
HP_ABNORMAL_JOINT_MORPHOLOGY	1151	Abnormal joint morphology	63	2.31E-20
HP_ABNORMALITY_OF_THE_CURVATURE_OF_THE_VERTEBRAL_COLUMN	1136	Abnormality of the curvature of the vertebral column	62	5.45E-20
HP_ABNORMALITY_OF_THE_OUTER_EAR	1230	Abnormality of the outer ear	64	1.03E-19
RODRIGUES_THYROID_CARCINOMA_POORLY_DIFFERENTIATED_DN	802	Genes down-regulated in poorly differentiated thyroid carcinoma (PDTC) compared to normal thyroid tissue.	52	1.03E-19
HP_ABNORMAL_NASAL_BRIDGE_MORPHOLOGY	1039	Abnormal nasal bridge morphology	58	3.92E-19

Table S15. Top ten most significantly enriched genesets of non-computational collections (C1, C2, C3, C5, C6, C7, C8, H) for the top 500 genes of miRNA-gene pairs with most significant corrected p-values in ILC setting TBN.

Geneset Name	# Genes in Geneset	Description	# Genes in Overlap	FDR q-value
GRAESSMANN_APOPTOSIS_BY_DOXORUBICIN_DN	1775	Genes down-regulated in ME-A cells (breast cancer) undergoing apoptosis in response to doxorubicin [PubChem=31703].	89	7.61E-27
NAKAYA_PBMC_FLUMIST_AGE_18_50YO_7DY_UP	1575	Genes up-regulated in peripheral blood mononuclear cell 7d vs 0d in adults (18-50) after exposure to FluMist , time point 7D. Comment: Supplementary Table 1b: All the differentially expressed genes identified in PBMCs of TIV vaccinees.	75	8.56E-21
DIAZ_CHRONIC_MYELOGENOUS_LEUKEMIA_UP	1399	Genes up-regulated in CD34+ [GeneID=947] cells isolated from bone marrow of CML (chronic myelogenous leukemia) patients, compared to those from normal donors.	67	1.83E-18
GOMF_RNA_BINDING	1968	Binding to an RNA molecule or a portion thereof. [GOC:jl, GOC:mah]	80	2.05E-18
BLALOCK_ALZHEIMERS_DISEASE_UP	1671	Genes up-regulated in brain from patients with Alzheimer's disease.	73	2.4E-18
GOBP_CELL_PROJECTION_ORGANIZATION	1576	A process that is carried out at the cellular level which results in the assembly, arrangement of constituent parts, or disassembly of a prolongation or process extending from a cell, e.g. a flagellum or axon. [GOC:jl, GOC:mah, http://www.cogsci.princeton.edu/ wn/]	70	6.34E-18
GOBP_POSITIVE_REGULATION_OF_CELLULAR_COMPONENT_ORGANIZATION	1068	Any process that activates or increases the frequency, rate or extent of a process involved in the formation, arrangement of constituent parts, or disassembly of cell structures, including the plasma membrane and any external encapsulating structures such as the cell wall and cell envelope. [GOC:ai]	57	9.35E-18
SALL4_TARGET_GENES	1870	Genes containing one or more binding sites for UniProt:Q9UJQ4 (SALL4) in their promoter regions (TSS -1000,+100 bp) as identified by GTRD version 20.06 ChIP-seq harmonization.	76	1.34E-17
HP_ABNORMAL_CARDIAC_SEPTUM_MORPHOLOGY	734	Abnormal cardiac septum morphology	47	2.07E-17
NAKAYA_PLASMACYTOID_DENDRITIC_CELL_FLUMIST_AGE_18_50YO_7DY_UP	1215	Genes up-regulated in plasmacytoid dendritic cell 7d vs 0d in young adults (18-50) after exposure to FluMist , time point 7D	60	2.44E-17

Table S16. Top ten most significantly enriched genesets of non-computational collections (C1, C2, C3, C5, C6, C7, C8, H) for the top 500 genes of miRNA-gene pairs with most significant corrected p-values in ILC setting ALLT.

Geneset Name	# Genes in Geneset	Description	# Genes in Overlap	FDR q-value
DIAZ_CHRONIC_MYELOGENOUS_LEUKEMIA_UP	1399	Genes up-regulated in CD34+ [GeneID=947] cells isolated from bone marrow of CML (chronic myelogenous leukemia) patients, compared to those from normal donors.	69	1.62E-19
PUJANA_BRCA1_PCC_NETWORK	1625	Genes constituting the BRCA1-PCC network of transcripts whose expression positively correlated (Pearson correlation coefficient, PCC >= 0.4) with that of BRCA1 [GeneID=672] across a compendium of normal tissues.	73	7.9E-19
HE_LIM_SUN_FETAL_LUNG_C2_HSC_CELL	1635	HSC	73	7.9E-19
GOMF_RNA_BINDING	1968	Binding to an RNA molecule or a portion thereof. [GOC:jl, GOC:mah]	80	1.39E-18
GOBP_INTRACELLULAR_TRANSPORT	1591	The directed movement of substances within a cell. [GOC:ai]	71	2.03E-18
GRAESSMANN_APOPTOSIS_BY_DOXORUBICIN_DN	1775	Genes down-regulated in ME-A cells (breast cancer) undergoing apoptosis in response to doxorubicin [PubChem=31703].	73	4.33E-17
NAKAYA_PBMC_FLUMIST_AGE_18_50YO_7DY_UP	1575	Genes up-regulated in peripheral blood mononuclear cell 7d vs 0d in adults (18-50) after exposure to FluMist , time point 7D. Comment: Supplementary Table 1b: All the differentially expressed genes identified in PBMCs of TIV vaccinees.	67	3.04E-16
BUSSLINGER_GASTRIC_IMMUNE_CELLS	1492	-	65	3.04E-16
GOCC_NUCLEAR_PROTEIN_CONTAINING_COMPLEX	1243	A stable assembly of two or more macromolecules, i.e. proteins, nucleic acids, carbohydrates or lipids, in which at least one component is a protein and the constituent parts function together in the nucleus. [GOC:pg]	58	1.36E-15
ACEVEDO_LIVER_CANCER_UP	972	Genes up-regulated in hepatocellular carcinoma (HCC) compared to normal liver samples.	49	3.96E-14

Table S17. Top ten most significantly enriched genesets of non-computational collections (C1, C2, C3, C5, C6, C7, C8, H) for the top 500 genes of miRNA-gene pairs with most significant corrected p-values in IDC setting TNBN.

Geneset Name	# Genes in Geneset	Description	# Genes in Overlap	FDR q-value
SMID_BREAST_CANCER_BASAL_DN	699	Genes down-regulated in basal subtype of breast cancer samles.	76	3.98E-45
TRAVAGLINI_LUNG_PROXIMAL_CILIATED_CELL	1770	-	89	3.62E-27
FARMER_BREAST_CANCER_BASAL_VS_LULMINAL	329	Genes which best discriminated between two groups of breast cancer according to the status of ESR1 and AR [GeneID=2099;367]: basal (ESR1- AR-) and luminal (ESR1+ AR+).	40	3.21E-24
CHARAFE_BREAST_CANCER_LUMINAL_VS_BASAL_UP	384	Genes up-regulated in luminal-like breast cancer cell lines compared to the basal-like ones.	42	7.73E-24
CHARAFE_BREAST_CANCER_LUMINAL_VS_MESENCHYMAL_UP	454	Genes up-regulated in luminal-like breast cancer cell lines compared to the mesenchymal-like ones.	44	5.17E-23
DODD_NASOPHARYNGEAL_CARCINOMA_UP	1813	Genes up-regulated in nasopharyngeal carcinoma (NPC) compared to the normal tissue.	81	2.05E-21
GRAESSMANN_APOPTOSIS_BY_DOXORUBICIN_DN	1775	Genes down-regulated in ME-A cells (breast cancer) undergoing apoptosis in response to doxorubicin [PubChem=31703].	77	1.85E-19
NFE2L1_TARGET_GENES	1913	Genes containing one or more binding sites for UniProt:Q14494 (NFE2L1) in their promoter regions (TSS -1000,+100 bp) as identified by GTRD version 20.06 ChIP-seq harmonization.	80	2.02E-19
VANTVEER_BREAST_CANCER_ESR1_UP	149	Up-regulated genes from the optimal set of 550 markers discriminating breast cancer samples by ESR1 [GeneID=2099] expression: ER(+) vs ER(-) tumors.	26	2.53E-19
BRCA2_TARGET_GENES	1674	Genes containing one or more binding sites for UniProt:P51587 (BRCA2) in their promoter regions (TSS -1000,+100 bp) as identified by GTRD version 20.06 ChIP-seq harmonization.	73	1.5E-18

Table S18. Top ten most significantly enriched genesets of non-computational collections (C1, C2, C3, C5, C6, C7, C8, H) for the top 500 genes of miRNA-gene pairs with most significant corrected p-values in IDC setting TBN.

Geneset Name	# Genes in Geneset	Description	# Genes in Overlap	FDR q-value
SMID_BREAST_CANCER_BASAL_DN	699	Genes down-regulated in basal subtype of breast cancer samles.	71	7.99E-40
RODRIGUES_THYROID_CARCINOMA_POORLY_DIFFERENTIATED_DN	802	Genes down-regulated in poorly differentiated thyroid carcinoma (PDTC) compared to normal thyroid tissue.	57	2.83E-23
GRAESSMANN_APOPTOSIS_BY_DOXORUBICIN_DN	1775	Genes down-regulated in ME-A cells (breast cancer) undergoing apoptosis in response to doxorubicin [PubChem=31703].	80	4.82E-21
CHARAFE_BREAST_CANCER_LUMINAL_VS_BASAL_UP	384	Genes up-regulated in luminal-like breast cancer cell lines compared to the basal-like ones.	36	9.58E-18
NAKAYA_PLASMACYTOID_DENDRITIC_CELL_FLUMIST_AGE_18_50YO_7DY_UP	1215	Genes up-regulated in plasmacytoid dendritic cell 7d vs 0d in young adults (18-50) after exposure to FluMist , time point 7D	59	2.62E-16
SCGGAAGY_ELK1_02	1242	Genes having at least one occurrence of the highly conserved motif M3 SCGGAAGY in the regions spanning 4 kb centered on their transcription starting sites [-2kb, +2kb]. This matches the ELK1 [GeneSymbol=ELK1] transcription factor binding site V$ELK1_02 (v7.4 TRANSFAC).	59	6.2E-16
ZNF407_TARGET_GENES	1996	Genes containing one or more binding sites for UniProt:Q9C0G0 (ZNF407) in their promoter regions (TSS -1000,+100 bp) as identified by GTRD version 20.06 ChIP-seq harmonization.	76	7.42E-16
TGGAAA_NFAT_Q4_01	1935	Genes having at least one occurrence of the highly conserved motif M55 TGGAAA in the regions spanning 4 kb centered on their transcription starting sites [-2kb, +2kb]. This matches the NFAT [GeneSymbol=NFAT], NFATC [GeneSymbol=NFATC] transcription factor binding site V$NFAT_Q4_01 (v7.4 TRANSFAC).	74	1.54E-15
KRIGE_RESPONSE_TO_TOSEDOSTAT_24HR_UP	780	Genes up-regulated in HL-60 cells (acute promyelocytic leukemia, APL) after treatment with the aminopeptidase inhibitor tosedostat (CHR-2797) [PubChem=15547703] for 24 h.	46	1.54E-15
ZNF711_TARGET_GENES	1762	Genes containing one or more binding sites for UniProt:Q9Y462 (ZNF711) in their promoter regions (TSS -1000,+100 bp) as identified by GTRD version 20.06 ChIP-seq harmonization.	70	1.81E-15

Table S19. Top ten most significantly enriched genesets of non-computational collections (C1, C2, C3, C5, C6, C7, C8, H) for the top 500 genes of miRNA-gene pairs with most significant corrected p-values in IDC setting ALLT.

Geneset Name	# Genes in Geneset	Description	# Genes in Overlap	FDR q-value
SMID_BREAST_CANCER_BASAL_DN	699	Genes down-regulated in basal subtype of breast cancer samles.	74	4.14E-43
CHARAFE_BREAST_CANCER_LUMINAL_VS_MESENCHYMAL_UP	454	Genes up-regulated in luminal-like breast cancer cell lines compared to the mesenchymal-like ones.	61	3.24E-41
VANTVEER_BREAST_CANCER_ESR1_UP	149	Up-regulated genes from the optimal set of 550 markers discriminating breast cancer samples by ESR1 [GeneID=2099] expression: ER(+) vs ER(-) tumors.	35	3.35E-31
FARMER_BREAST_CANCER_BASAL_VS_LULMINAL	329	Genes which best discriminated between two groups of breast cancer according to the status of ESR1 and AR [GeneID=2099;367]: basal (ESR1- AR-) and luminal (ESR1+ AR+).	44	6.09E-29
TRAVAGLINI_LUNG_PROXIMAL_CILIATED_CELL	1770	-	89	1.06E-27
CHARAFE_BREAST_CANCER_LUMINAL_VS_BASAL_UP	384	Genes up-regulated in luminal-like breast cancer cell lines compared to the basal-like ones.	45	2.63E-27
DODD_NASOPHARYNGEAL_CARCINOMA_UP	1813	Genes up-regulated in nasopharyngeal carcinoma (NPC) compared to the normal tissue.	79	2.71E-20
LIM_MAMMARY_STEM_CELL_DN	416	Genes consistently down-regulated in mammary stem cells both in mouse and human species.	37	6.88E-18
FXR1_TARGET_GENES	1217	Genes containing one or more binding sites for UniProt:P51114 (FXR1) in their promoter regions (TSS -1000,+100 bp) as identified by GTRD version 20.06 ChIP-seq harmonization.	58	6.13E-16
RODRIGUES_THYROID_CARCINOMA_POORLY_DIFFERENTIATED_DN	802	Genes down-regulated in poorly differentiated thyroid carcinoma (PDTC) compared to normal thyroid tissue.	47	6.13E-16

Table S20. Top ten most significantly enriched genesets of oncogenic collection (C6) for the top 500 genes of miRNA-gene pairs with most significant corrected p-values in LGG setting TNBN.

Geneset Name	# Genes in Geneset	Description	# Genes in Overlap	FDR q-value
KRAS.KIDNEY_UP.V1_UP	141	Genes up-regulated in epithelial kidney cancer cell lines over-expressing an oncogenic form of KRAS [Gene ID=3845] gene.	15	2.77E-8
TBK1.DF_DN	286	Genes down-regulated in epithelial lung cancer cell lines upon over-expression of an oncogenic form of KRAS [Gene ID=3845] gene and knockdown of TBK1 [Gene ID=29110] gene by RNAi.	17	6.24E-6
TBK1.DN.48HRS_UP	50	Genes up-regulated in epithelial lung cancer cell lines upon over-expression of an oncogenic form of KRAS [Gene ID=3845] gene and knockdown of TBK1 [Gene ID=29110] gene by RNAi.	6	1.34E-3
CAMP_UP.V1_UP	200	Genes up-regulated in primary thyrocyte cultures in response to cAMP signaling pathway activation by thyrotropin (TSH).	11	1.34E-3
MTOR_UP.V1_DN	182	Genes down-regulated by everolimus [PubChem = 6442177] in prostate tissue.	10	2.48E-3
KRAS.600_UP.V1_UP	278	Genes up-regulated in four lineages of epithelial cell lines over-expressing an oncogenic form of KRAS [Gene ID=3845] gene.	12	4.06E-3
KRAS.300_UP.V1_UP	142	Genes up-regulated in four lineages of epithelial cell lines over-expressing an oncogenic form of KRAS [Gene ID=3845] gene.	8	7.95E-3
RB_P130_DN.V1_UP	130	Genes up-regulated in primary keratinocytes from RB1 and RBL2 [Gene ID=5925, 5934] skin specific knockout mice.	7	1.94E-2
SIRNA_EIF4GI_UP	95	Genes up-regulated in MCF10A cells vs knockdown of EIF4G1 [Gene ID=1981] gene by RNAi.	6	1.94E-2
AKT_UP.V1_DN	187	Genes down-regulated in mouse prostate by transgenic expression of human AKT1 gene [Gene ID=207] vs controls.	8	3.07E-2

Table S21. Top ten most significantly enriched genesets of oncogenic collection (C6) for the top 500 genes of miRNA-gene pairs with most significant corrected p-values in LGG setting TBN.

Geneset Name	# Genes in Geneset	Description	# Genes in Overlap	FDR q-value
CAMP_UP.V1_UP	200	Genes up-regulated in primary thyrocyte cultures in response to cAMP signaling pathway activation by thyrotropin (TSH).	16	4.55E-7
LTE2_UP.V1_DN	195	Genes down-regulated in MCF-7 cells (breast cancer) positive for ESR1 [Gene ID=2099] MCF-7 cells (breast cancer) and long-term adapted for estrogen-independent growth.	12	3E-4
CAMP_UP.V1_DN	199	Genes down-regulated in primary thyrocyte cultures in response to cAMP signaling pathway activation by thyrotropin (TSH).	12	3E-4
TBK1.DF_DN	286	Genes down-regulated in epithelial lung cancer cell lines upon over-expression of an oncogenic form of KRAS [Gene ID=3845] gene and knockdown of TBK1 [Gene ID=29110] gene by RNAi.	14	4.17E-4
KRAS.KIDNEY_UP.V1_UP	141	Genes up-regulated in epithelial kidney cancer cell lines over-expressing an oncogenic form of KRAS [Gene ID=3845] gene.	9	1.55E-3
NFE2L2.V2	469	Genes down-regulated in MEF cells (embryonic fibroblasts) after knockout of NFE2L2 [Gene ID=4780] gene.	17	1.55E-3
RB_DN.V1_UP	116	Genes up-regulated in primary keratinocytes from RB1 [Gene ID=5925] skin specific knockout mice.	8	1.94E-3
RAF_UP.V1_DN	193	Genes down-regulated in MCF-7 cells (breast cancer) positive for ESR1 [Gene ID=2099] MCF-7 cells (breast cancer) stably over-expressing constitutively active RAF1 [Gene ID=5894] gene.	10	2.48E-3
BMI1_DN_MEL18_DN.V1_UP	145	Genes up-regulated in DAOY cells (medulloblastoma) upon knockdown of BMI1 and PCGF2 [Gene ID=648, 7703] genes by RNAi.	8	7.02E-3
E2F1_UP.V1_DN	187	Genes down-regulated in mouse fibroblasts over-expressing E2F1 [Gene ID=1869] gene.	9	7.5E-3

Table S22. Top ten most significantly enriched genesets of oncogenic collection (C6) for the top 500 genes of miRNA-gene pairs with most significant corrected p-values in LGG setting ALLT.

Geneset Name	# Genes in Geneset	Description	# Genes in Overlap	FDR q-value
CAMP_UP.V1_DN	199	Genes down-regulated in primary thyrocyte cultures in response to cAMP signaling pathway activation by thyrotropin (TSH).	11	2.53E-3
CAMP_UP.V1_UP	200	Genes up-regulated in primary thyrocyte cultures in response to cAMP signaling pathway activation by thyrotropin (TSH).	11	2.53E-3
KRAS.KIDNEY_UP.V1_UP	141	Genes up-regulated in epithelial kidney cancer cell lines over-expressing an oncogenic form of KRAS [Gene ID=3845] gene.	9	2.87E-3
TBK1.DF_DN	286	Genes down-regulated in epithelial lung cancer cell lines upon over-expression of an oncogenic form of KRAS [Gene ID=3845] gene and knockdown of TBK1 [Gene ID=29110] gene by RNAi.	12	7.51E-3
RB_P130_DN.V1_DN	136	Genes down-regulated in primary keratinocytes from RB1 and RBL2 [Gene ID=5925, 5934] skin specific knockout mice.	8	7.97E-3
MTOR_UP.V1_DN	182	Genes down-regulated by everolimus [PubChem = 6442177] in prostate tissue.	9	9.84E-3
E2F1_UP.V1_DN	187	Genes down-regulated in mouse fibroblasts over-expressing E2F1 [Gene ID=1869] gene.	9	9.84E-3
TGFB_UP.V1_UP	189	Genes up-regulated in a panel of epithelial cell lines by TGFB1 [Gene ID=7040].	9	9.84E-3
SIRNA_EIF4GI_UP	95	Genes up-regulated in MCF10A cells vs knockdown of EIF4G1 [Gene ID=1981] gene by RNAi.	6	1.88E-2
EIF4E_UP	100	Genes up-regulated in HMEC cells (primary mammary epithelium) upon over-expression of EIF4E [Gene ID=1977] gene.	6	2.21E-2

Table S23. Top ten most significantly enriched genesets of oncogenic collection (C6) for the top 500 genes of miRNA-gene pairs with most significant corrected p-values in LIHC setting TNBN.

Geneset Name	# Genes in Geneset	Description	# Genes in Overlap	FDR q-value
CYCLIN_D1_KE_.V1_UP	188	Genes up-regulated in MCF-7 cells (breast cancer) over-expressing a mutant K112E form of CCND1 [Gene ID=595] gene.	15	1.53E-6
SIRNA_EIF4GI_UP	95	Genes up-regulated in MCF10A cells vs knockdown of EIF4G1 [Gene ID=1981] gene by RNAi.	8	1.63E-3
NFE2L2.V2	469	Genes down-regulated in MEF cells (embryonic fibroblasts) after knockout of NFE2L2 [Gene ID=4780] gene.	17	3.18E-3
HOXA9_DN.V1_DN	190	Genes down-regulated in MOLM-14 cells (AML) with knockdown of HOXA9 [Gene ID=3205] gene by RNAi vs controls.	10	4.43E-3
RB_P107_DN.V1_UP	130	Genes up-regulated in primary keratinocytes from RB1 and RBL1 [Gene ID=5925, 5933] skin specific knockout mice.	8	5.88E-3
EIF4E_UP	100	Genes up-regulated in HMEC cells (primary mammary epithelium) upon over-expression of EIF4E [Gene ID=1977] gene.	7	5.88E-3
CYCLIN_D1_UP.V1_UP	187	Genes up-regulated in MCF-7 cells (breast cancer) over-expressing CCND1 [Gene ID=595] gene.	9	9.49E-3
E2F1_UP.V1_UP	188	Genes up-regulated in mouse fibroblasts over-expressing E2F1 [Gene ID=1869] gene.	9	9.49E-3
CYCLIN_D1_UP.V1_DN	190	Genes down-regulated in MCF-7 cells (breast cancer) over-expressing CCND1 [Gene ID=595] gene.	9	9.49E-3
MTOR_UP.N4.V1_UP	196	Genes up-regulated in CEM-C1 cells (T-CLL) in comparison of control vs rapamycin (sirolimus) [PubChem=6610346], an mTOR pathway inhibitor.	9	1.07E-2

Table S24. Top ten most significantly enriched genesets of oncogenic collection (C6) for the top 500 genes of miRNA-gene pairs with most significant corrected p-values in LIHC setting TBN.

Geneset Name	# Genes in Geneset	Description	# Genes in Overlap	FDR q-value
ATF2_UP.V1_DN	185	Genes down-regulated in myometrial cells over-expressing ATF2 [Gene ID=1386] gene.	15	6.92E-7
ATF2_S_UP.V1_DN	187	Genes down-regulated in myometrial cells over-expressing a shortened splice form of ATF2 [Gene ID=1386] gene.	15	6.92E-7
MTOR_UP.N4.V1_UP	196	Genes up-regulated in CEM-C1 cells (T-CLL) in comparison of control vs rapamycin (sirolimus) [PubChem=6610346], an mTOR pathway inhibitor.	15	8.72E-7
RPS14_DN.V1_DN	186	Genes down-regulated in CD34+ hematopoietic progenitor cells after knockdown of RPS14 [Gene ID=6208] by RNAi.	14	2.24E-6
CYCLIN_D1_KE_.V1_UP	188	Genes up-regulated in MCF-7 cells (breast cancer) over-expressing a mutant K112E form of CCND1 [Gene ID=595] gene.	14	2.24E-6
RAPA_EARLY_UP.V1_DN	186	Genes down-regulated in BJAB (lymphoma) cells by rapamycin (sirolimus) [PubChem = 6610346].	13	1.11E-5
LTE2_UP.V1_UP	188	Genes up-regulated in MCF-7 cells (breast cancer) positive for ESR1 [Gene ID=2099] MCF-7 cells (breast cancer) and long-term adapted for estrogen-independent growth.	13	1.11E-5
RAF_UP.V1_UP	193	Genes up-regulated in MCF-7 cells (breast cancer) positive for ESR1 [Gene ID=2099] MCF-7 cells (breast cancer) stably over-expressing constitutively active RAF1 [Gene ID=5894] gene.	13	1.31E-5
CYCLIN_D1_UP.V1_UP	187	Genes up-regulated in MCF-7 cells (breast cancer) over-expressing CCND1 [Gene ID=595] gene.	11	3.12E-4
SIRNA_EIF4GI_UP	95	Genes up-regulated in MCF10A cells vs knockdown of EIF4G1 [Gene ID=1981] gene by RNAi.	8	3.12E-4

Table S25. Top ten most significantly enriched genesets of oncogenic collection (C6) for the top 500 genes of miRNA-gene pairs with most significant corrected p-values in LIHC setting ALLT.

Geneset Name	# Genes in Geneset	Description	# Genes in Overlap	FDR q-value
CYCLIN_D1_KE_.V1_UP	188	Genes up-regulated in MCF-7 cells (breast cancer) over-expressing a mutant K112E form of CCND1 [Gene ID=595] gene.	14	1.01E-5
MYC_UP.V1_UP	181	Genes up-regulated in primary epithelial breast cancer cell culture over-expressing MYC [Gene ID=4609] gene.	13	2.3E-5
E2F1_UP.V1_UP	188	Genes up-regulated in mouse fibroblasts over-expressing E2F1 [Gene ID=1869] gene.	12	1.54E-4
SIRNA_EIF4GI_UP	95	Genes up-regulated in MCF10A cells vs knockdown of EIF4G1 [Gene ID=1981] gene by RNAi.	8	7.58E-4
GCNP_SHH_UP_EARLY.V1_UP	174	Genes up-regulated in granule cell neuron precursors (GCNPs) after stimulation with Shh for 3h.	9	8.32E-3
MTOR_UP.N4.V1_UP	196	Genes up-regulated in CEM-C1 cells (T-CLL) in comparison of control vs rapamycin (sirolimus) [PubChem=6610346], an mTOR pathway inhibitor.	9	1.66E-2
RB_P107_DN.V1_UP	130	Genes up-regulated in primary keratinocytes from RB1 and RBL1 [Gene ID=5925, 5933] skin specific knockout mice.	7	2.32E-2
ESC_J1_UP_LATE.V1_UP	185	Genes up-regulated during late stages of differentiation of embryoid bodies from J1 embryonic stem cells.	8	3.04E-2
LTE2_UP.V1_UP	188	Genes up-regulated in MCF-7 cells (breast cancer) positive for ESR1 [Gene ID=2099] MCF-7 cells (breast cancer) and long-term adapted for estrogen-independent growth.	8	3.04E-2
HOXA9_DN.V1_DN	190	Genes down-regulated in MOLM-14 cells (AML) with knockdown of HOXA9 [Gene ID=3205] gene by RNAi vs controls.	8	3.04E-2

Table S26. Top ten most significantly enriched genesets of oncogenic collection (C6) for the top 500 genes of miRNA-gene pairs with most significant corrected p-values in KIRC setting TNBN.

Geneset Name	# Genes in Geneset	Description	# Genes in Overlap	FDR q-value
VEGF_A_UP.V1_UP	195	Genes up-regulated in HUVEC cells (endothelium) by treatment with VEGFA [Gene ID=7422].	17	3.8E-8
ERBB2_UP.V1_UP	190	Genes up-regulated in MCF-7 cells (breast cancer) positive for ESR1 [Gene ID=2099] and engineered to express ligand-activatable ERBB2 [Gene ID=2064].	15	8.59E-7
STK33_UP	286	Genes up-regulated in NOMO-1 and SKM-1 cells (AML) after knockdown of STK33 [Gene ID=65975] by RNAi.	17	3.41E-6
RPS14_DN.V1_UP	191	Genes up-regulated in CD34+ hematopoietic progenitor cells after knockdown of RPS14 [Gene ID=6208] by RNAi.	14	3.41E-6
BMI1_DN_MEL18_DN.V1_UP	145	Genes up-regulated in DAOY cells (medulloblastoma) upon knockdown of BMI1 and PCGF2 [Gene ID=648, 7703] genes by RNAi.	12	6.27E-6
TBK1.DF_DN	286	Genes down-regulated in epithelial lung cancer cell lines upon over-expression of an oncogenic form of KRAS [Gene ID=3845] gene and knockdown of TBK1 [Gene ID=29110] gene by RNAi.	15	5.3E-5
STK33_NOMO_UP	290	Genes up-regulated in NOMO-1 cells (AML) after knockdown of STK33 [Gene ID=65975] by RNAi.	15	5.3E-5
ESC_J1_UP_LATE.V1_UP	185	Genes up-regulated during late stages of differentiation of embryoid bodies from J1 embryonic stem cells.	12	5.3E-5
CYCLIN_D1_KE_.V1_DN	191	Genes down-regulated in MCF-7 cells (breast cancer) over-expressing a mutant K112E form of CCND1 [Gene ID=595] gene.	12	5.91E-5
KRAS.DF.V1_UP	191	Genes up-regulated in epithelial lung cancer cell lines over-expressing an oncogenic form of KRAS [Gene ID=3845] gene.	12	5.91E-5

Table S27. Top ten most significantly enriched genesets of oncogenic collection (C6) for the top 500 genes of miRNA-gene pairs with most significant corrected p-values in KIRC setting TBN.

Geneset Name	# Genes in Geneset	Description	# Genes in Overlap	FDR q-value
LTE2_UP.V1_UP	188	Genes up-regulated in MCF-7 cells (breast cancer) positive for ESR1 [Gene ID=2099] MCF-7 cells (breast cancer) and long-term adapted for estrogen-independent growth.	15	1.49E-6
RAF_UP.V1_DN	193	Genes down-regulated in MCF-7 cells (breast cancer) positive for ESR1 [Gene ID=2099] MCF-7 cells (breast cancer) stably over-expressing constitutively active RAF1 [Gene ID=5894] gene.	13	5.26E-5
CAHOY_ASTROCYTIC	100	Genes up-regulated in astrocytes.	9	1.84E-4
ESC_V6.5_UP_LATE.V1_UP	184	Genes up-regulated during late stages of differentiation of embryoid bodies from V6.5 embryonic stem cells.	11	6.05E-4
EGFR_UP.V1_DN	196	Genes down-regulated in MCF-7 cells (breast cancer) positive for ESR1 [Gene ID=2099] and engineered to express ligand-activatable EGFR [Gene ID=1956].	11	8.36E-4
CAMP_UP.V1_DN	199	Genes down-regulated in primary thyrocyte cultures in response to cAMP signaling pathway activation by thyrotropin (TSH).	11	8.36E-4
TBK1.DF_DN	286	Genes down-regulated in epithelial lung cancer cell lines upon over-expression of an oncogenic form of KRAS [Gene ID=3845] gene and knockdown of TBK1 [Gene ID=29110] gene by RNAi.	13	1.07E-3
ESC_V6.5_UP_LATE.V1_DN	180	Genes down-regulated during late stages of differentiation of embryoid bodies from V6.5 embryonic stem cells.	10	1.29E-3
JAK2_DN.V1_DN	146	Genes down-regulated in HEL cells (erythroleukemia) after knockdown of JAK2 [Gene ID=3717] gene by RNAi.	9	1.29E-3
E2F1_UP.V1_DN	187	Genes down-regulated in mouse fibroblasts over-expressing E2F1 [Gene ID=1869] gene.	10	1.53E-3

Table S28. Top ten most significantly enriched genesets of oncogenic collection (C6) for the top 500 genes of miRNA-gene pairs with most significant corrected p-values in KIRC setting ALLT.

Geneset Name	# Genes in Geneset	Description	# Genes in Overlap	FDR q-value
BMI1_DN.V1_UP	147	Genes up-regulated in DAOY cells (medulloblastoma) upon knockdown of BMI1 [Gene ID=648] gene by RNAi.	13	4.25E-6
ERBB2_UP.V1_UP	190	Genes up-regulated in MCF-7 cells (breast cancer) positive for ESR1 [Gene ID=2099] and engineered to express ligand-activatable ERBB2 [Gene ID=2064].	14	5.05E-6
RAF_UP.V1_UP	193	Genes up-regulated in MCF-7 cells (breast cancer) positive for ESR1 [Gene ID=2099] MCF-7 cells (breast cancer) stably over-expressing constitutively active RAF1 [Gene ID=5894] gene.	14	5.05E-6
BMI1_DN_MEL18_DN.V1_UP	145	Genes up-regulated in DAOY cells (medulloblastoma) upon knockdown of BMI1 and PCGF2 [Gene ID=648, 7703] genes by RNAi.	12	7.67E-6
ESC_V6.5_UP_LATE.V1_UP	184	Genes up-regulated during late stages of differentiation of embryoid bodies from V6.5 embryonic stem cells.	12	7.64E-5
LTE2_UP.V1_UP	188	Genes up-regulated in MCF-7 cells (breast cancer) positive for ESR1 [Gene ID=2099] MCF-7 cells (breast cancer) and long-term adapted for estrogen-independent growth.	12	7.64E-5
CYCLIN_D1_KE_.V1_DN	191	Genes down-regulated in MCF-7 cells (breast cancer) over-expressing a mutant K112E form of CCND1 [Gene ID=595] gene.	12	7.64E-5
EGFR_UP.V1_UP	192	Genes up-regulated in MCF-7 cells (breast cancer) positive for ESR1 [Gene ID=2099] and engineered to express ligand-activatable EGFR [Gene ID=1956].	12	7.64E-5
CRX_DN.V1_UP	135	Genes up-regulated in retina cells from CRX [Gene ID=1406] knockout mice.	10	9.99E-5
MTOR_UP.V1_UP	169	Genes up-regulated by everolimus [PubChem = 6442177] in prostate tissue.	11	1.06E-4

Table S29. Top ten most significantly enriched genesets of oncogenic collection (C6) for the top 500 genes of miRNA-gene pairs with most significant corrected p-values in KICH setting TNBN.

Geneset Name	# Genes in Geneset	Description	# Genes in Overlap	FDR q-value
CAMP_UP.V1_DN	199	Genes down-regulated in primary thyrocyte cultures in response to cAMP signaling pathway activation by thyrotropin (TSH).	14	2.21E-5
CAMP_UP.V1_UP	200	Genes up-regulated in primary thyrocyte cultures in response to cAMP signaling pathway activation by thyrotropin (TSH).	13	7.7E-5
RAF_UP.V1_UP	193	Genes up-regulated in MCF-7 cells (breast cancer) positive for ESR1 [Gene ID=2099] MCF-7 cells (breast cancer) stably over-expressing constitutively active RAF1 [Gene ID=5894] gene.	11	1.12E-3
ERBB2_UP.V1_DN	197	Genes down-regulated in MCF-7 cells (breast cancer) positive for ESR1 [Gene ID=2099] and engineered to express ligand-activatable ERBB2 [Gene ID=2064].	11	1.12E-3
GCNP_SHH_UP_EARLY.V1_UP	174	Genes up-regulated in granule cell neuron precursors (GCNPs) after stimulation with Shh for 3h.	10	1.64E-3
GCNP_SHH_UP_LATE.V1_UP	181	Genes up-regulated in granule cell neuron precursors (GCNPs) after stimulation with Shh for 24h.	10	1.91E-3
ESC_J1_UP_LATE.V1_UP	185	Genes up-regulated during late stages of differentiation of embryoid bodies from J1 embryonic stem cells.	10	1.96E-3
ERBB2_UP.V1_UP	190	Genes up-regulated in MCF-7 cells (breast cancer) positive for ESR1 [Gene ID=2099] and engineered to express ligand-activatable ERBB2 [Gene ID=2064].	10	2.14E-3
P53_DN.V1_UP	194	Genes up-regulated in NCI-60 panel of cell lines with mutated TP53 [Gene ID=7157].	10	2.26E-3
SIRNA_EIF4GI_UP	95	Genes up-regulated in MCF10A cells vs knockdown of EIF4G1 [Gene ID=1981] gene by RNAi.	7	2.5E-3

Table S30. Top ten most significantly enriched genesets of oncogenic collection (C6) for the top 500 genes of miRNA-gene pairs with most significant corrected p-values in KICH setting TBN.

Geneset Name	# Genes in Geneset	Description	# Genes in Overlap	FDR q-value
RAF_UP.V1_UP	193	Genes up-regulated in MCF-7 cells (breast cancer) positive for ESR1 [Gene ID=2099] MCF-7 cells (breast cancer) stably over-expressing constitutively active RAF1 [Gene ID=5894] gene.	15	1.76E-6
CAMP_UP.V1_UP	200	Genes up-regulated in primary thyrocyte cultures in response to cAMP signaling pathway activation by thyrotropin (TSH).	15	1.76E-6
MEK_UP.V1_DN	194	Genes down-regulated in MCF-7 cells (breast cancer) positive for ESR1 [Gene ID=2099] MCF-7 cells (breast cancer) stably over-expressing constitutively active MAP2K1 [Gene ID=5604] gene.	12	1.97E-4
EGFR_UP.V1_DN	196	Genes down-regulated in MCF-7 cells (breast cancer) positive for ESR1 [Gene ID=2099] and engineered to express ligand-activatable EGFR [Gene ID=1956].	12	1.97E-4
LEF1_UP.V1_DN	187	Genes down-regulated in DLD1 cells (colon carcinoma) over-expressing LEF1 [Gene ID=51176].	11	5.55E-4
PGF_UP.V1_UP	190	Genes up-regulated in HUVEC cells (endothelium) by treatment with PGF [Gene ID=5281].	11	5.55E-4
CRX_NRL_DN.V1_DN	127	Genes down-regulated in retina cells from CRX and NRL [Gene ID=1406, 4901] double knockout mice.	9	5.64E-4
TBK1.DF_DN	286	Genes down-regulated in epithelial lung cancer cell lines upon over-expression of an oncogenic form of KRAS [Gene ID=3845] gene and knockdown of TBK1 [Gene ID=29110] gene by RNAi.	12	3.61E-3
NRL_DN.V1_DN	132	Genes down-regulated in retina cells from NRL [Gene ID=4901] knockout mice.	8	3.61E-3
STK33_NOMO_UP	290	Genes up-regulated in NOMO-1 cells (AML) after knockdown of STK33 [Gene ID=65975] by RNAi.	12	3.61E-3

Table S31. Top ten most significantly enriched genesets of oncogenic collection (C6) for the top 500 genes of miRNA-gene pairs with most significant corrected p-values in KICH setting ALLT.

Geneset Name	# Genes in Geneset	Description	# Genes in Overlap	FDR q-value
CAMP_UP.V1_UP	200	Genes up-regulated in primary thyrocyte cultures in response to cAMP signaling pathway activation by thyrotropin (TSH).	18	6.34E-9
ERBB2_UP.V1_UP	190	Genes up-regulated in MCF-7 cells (breast cancer) positive for ESR1 [Gene ID=2099] and engineered to express ligand-activatable ERBB2 [Gene ID=2064].	13	3.85E-5
MEK_UP.V1_UP	195	Genes up-regulated in MCF-7 cells (breast cancer) positive for ESR1 [Gene ID=2099] MCF-7 cells (breast cancer) stably over-expressing constitutively active MAP2K1 [Gene ID=5604] gene.	13	3.85E-5
LEF1_UP.V1_DN	187	Genes down-regulated in DLD1 cells (colon carcinoma) over-expressing LEF1 [Gene ID=51176].	12	1.16E-4
GCNP_SHH_UP_EARLY.V1_UP	174	Genes up-regulated in granule cell neuron precursors (GCNPs) after stimulation with Shh for 3h.	10	1.64E-3
GCNP_SHH_UP_LATE.V1_UP	181	Genes up-regulated in granule cell neuron precursors (GCNPs) after stimulation with Shh for 24h.	10	1.91E-3
PGF_UP.V1_UP	190	Genes up-regulated in HUVEC cells (endothelium) by treatment with PGF [Gene ID=5281].	10	2.45E-3
KRAS.300_UP.V1_DN	140	Genes down-regulated in four lineages of epithelial cell lines over-expressing an oncogenic form of KRAS [Gene ID=3845] gene.	8	6.15E-3
E2F1_UP.V1_UP	188	Genes up-regulated in mouse fibroblasts over-expressing E2F1 [Gene ID=1869] gene.	9	8.54E-3
VEGF_A_UP.V1_UP	195	Genes up-regulated in HUVEC cells (endothelium) by treatment with VEGFA [Gene ID=7422].	9	9.79E-3

Table S32. Top ten most significantly enriched genesets of oncogenic collection (C6) for the top 500 genes of miRNA-gene pairs with most significant corrected p-values in ILC setting TNBN.

Geneset Name	# Genes in Geneset	Description	# Genes in Overlap	FDR q-value
MEK_UP.V1_UP	195	Genes up-regulated in MCF-7 cells (breast cancer) positive for ESR1 [Gene ID=2099] MCF-7 cells (breast cancer) stably over-expressing constitutively active MAP2K1 [Gene ID=5604] gene.	15	2.51E-6
BMI1_DN.V1_UP	147	Genes up-regulated in DAOY cells (medulloblastoma) upon knockdown of BMI1 [Gene ID=648] gene by RNAi.	11	1.18E-4
E2F1_UP.V1_DN	187	Genes down-regulated in mouse fibroblasts over-expressing E2F1 [Gene ID=1869] gene.	12	1.18E-4
RB_P107_DN.V1_DN	126	Genes down-regulated in primary keratinocytes from RB1 and RBL1 [Gene ID=5925, 5933] skin specific knockout mice.	10	1.18E-4
MEK_UP.V1_DN	194	Genes down-regulated in MCF-7 cells (breast cancer) positive for ESR1 [Gene ID=2099] MCF-7 cells (breast cancer) stably over-expressing constitutively active MAP2K1 [Gene ID=5604] gene.	12	1.18E-4
P53_DN.V1_UP	194	Genes up-regulated in NCI-60 panel of cell lines with mutated TP53 [Gene ID=7157].	12	1.18E-4
GCNP_SHH_UP_LATE.V1_UP	181	Genes up-regulated in granule cell neuron precursors (GCNPs) after stimulation with Shh for 24h.	11	3.02E-4
E2F1_UP.V1_UP	188	Genes up-regulated in mouse fibroblasts over-expressing E2F1 [Gene ID=1869] gene.	11	3.77E-4
MEL18_DN.V1_UP	141	Genes up-regulated in DAOY cells (medulloblastoma) upon knockdown of PCGF2 [Gene ID=7703] gene by RNAi.	9	1E-3
GCNP_SHH_UP_LATE.V1_DN	178	Genes down-regulated in granule cell neuron precursors (GCNPs) after stimulation with Shh for 24h.	10	1.03E-3

Table S33. Top ten most significantly enriched genesets of oncogenic collection (C6) for the top 500 genes of miRNA-gene pairs with most significant corrected p-values in ILC setting TBN.

Geneset Name	# Genes in Geneset	Description	# Genes in Overlap	FDR q-value
TBK1.DF_DN	286	Genes down-regulated in epithelial lung cancer cell lines upon over-expression of an oncogenic form of KRAS [Gene ID=3845] gene and knockdown of TBK1 [Gene ID=29110] gene by RNAi.	14	1.38E-3
E2F1_UP.V1_DN	187	Genes down-regulated in mouse fibroblasts over-expressing E2F1 [Gene ID=1869] gene.	11	1.38E-3
EIF4E_DN	100	Genes down-regulated in HMEC cells (primary mammary epithelium) upon over-expression of EIF4E [Gene ID=1977] gene.	8	1.53E-3
CYCLIN_D1_UP.V1_DN	190	Genes down-regulated in MCF-7 cells (breast cancer) over-expressing CCND1 [Gene ID=595] gene.	10	3.9E-3
RAF_UP.V1_DN	193	Genes down-regulated in MCF-7 cells (breast cancer) positive for ESR1 [Gene ID=2099] MCF-7 cells (breast cancer) stably over-expressing constitutively active RAF1 [Gene ID=5894] gene.	10	3.9E-3
MTOR_UP.N4.V1_DN	184	Genes down-regulated in CEM-C1 cells (T-CLL) in comparison of control vs rapamycin (sirolimus) [PubChem=6610346], an mTOR pathway inhibitor.	9	9.65E-3
TGFB_UP.V1_UP	189	Genes up-regulated in a panel of epithelial cell lines by TGFB1 [Gene ID=7040].	9	9.65E-3
CYCLIN_D1_KE_.V1_DN	191	Genes down-regulated in MCF-7 cells (breast cancer) over-expressing a mutant K112E form of CCND1 [Gene ID=595] gene.	9	9.65E-3
PKCA_DN.V1_DN	154	Genes down-regulated in small intenstine in PRKCA [Gene ID=5578] knockout mice.	8	9.65E-3
LEF1_UP.V1_UP	194	Genes up-regulated in DLD1 cells (colon carcinoma) over-expressing LEF1 [Gene ID=51176].	9	9.65E-3

Table S34. Top ten most significantly enriched genesets of oncogenic collection (C6) for the top 500 genes of miRNA-gene pairs with most significant corrected p-values in ILC setting ALLT.

Geneset Name	# Genes in Geneset	Description	# Genes in Overlap	FDR q-value
TBK1.DF_UP	287	Genes up-regulated in epithelial lung cancer cell lines upon over-expression of an oncogenic form of KRAS [Gene ID=3845] gene and knockdown of TBK1 [Gene ID=29110] gene by RNAi.	18	1.93E-6
RAF_UP.V1_DN	193	Genes down-regulated in MCF-7 cells (breast cancer) positive for ESR1 [Gene ID=2099] MCF-7 cells (breast cancer) stably over-expressing constitutively active RAF1 [Gene ID=5894] gene.	13	4.53E-5
CAMP_UP.V1_DN	199	Genes down-regulated in primary thyrocyte cultures in response to cAMP signaling pathway activation by thyrotropin (TSH).	13	4.53E-5
STK33_UP	286	Genes up-regulated in NOMO-1 and SKM-1 cells (AML) after knockdown of STK33 [Gene ID=65975] by RNAi.	12	7.23E-3
BCAT_BILD_ET_AL_DN	46	Genes down-regulated in primary epithelial breast cancer cell culture over-expressing activated CTNNB1 [Gene ID=1499] gene.	5	7.34E-3
MTOR_UP.N4.V1_UP	196	Genes up-regulated in CEM-C1 cells (T-CLL) in comparison of control vs rapamycin (sirolimus) [PubChem=6610346], an mTOR pathway inhibitor.	9	1.66E-2
RB_P107_DN.V1_UP	130	Genes up-regulated in primary keratinocytes from RB1 and RBL1 [Gene ID=5925, 5933] skin specific knockout mice.	7	2.32E-2
GCNP_SHH_UP_EARLY.V1_UP	174	Genes up-regulated in granule cell neuron precursors (GCNPs) after stimulation with Shh for 3h.	8	2.49E-2
PGF_UP.V1_UP	190	Genes up-regulated in HUVEC cells (endothelium) by treatment with PGF [Gene ID=5281].	8	3.14E-2
HOXA9_DN.V1_UP	192	Genes up-regulated in MOLM-14 cells (AML) with knockdown of HOXA9 [Gene ID=3205] gene by RNAi vs controls.	8	3.14E-2

Table S35. Top ten most significantly enriched genesets of oncogenic collection (C6) for the top 500 genes of miRNA-gene pairs with most significant corrected p-values in IDC setting TNBN.

Geneset Name	# Genes in Geneset	Description	# Genes in Overlap	FDR q-value
HOXA9_DN.V1_UP	192	Genes up-regulated in MOLM-14 cells (AML) with knockdown of HOXA9 [Gene ID=3205] gene by RNAi vs controls.	13	5.26E-5
RAF_UP.V1_DN	193	Genes down-regulated in MCF-7 cells (breast cancer) positive for ESR1 [Gene ID=2099] MCF-7 cells (breast cancer) stably over-expressing constitutively active RAF1 [Gene ID=5894] gene.	13	5.26E-5
SIRNA_EIF4GI_UP	95	Genes up-regulated in MCF10A cells vs knockdown of EIF4G1 [Gene ID=1981] gene by RNAi.	9	1.2E-4
MTOR_UP.N4.V1_DN	184	Genes down-regulated in CEM-C1 cells (T-CLL) in comparison of control vs rapamycin (sirolimus) [PubChem=6610346], an mTOR pathway inhibitor.	11	6.05E-4
ERBB2_UP.V1_UP	190	Genes up-regulated in MCF-7 cells (breast cancer) positive for ESR1 [Gene ID=2099] and engineered to express ligand-activatable ERBB2 [Gene ID=2064].	11	6.54E-4
GCNP_SHH_UP_EARLY.V1_DN	167	Genes down-regulated in granule cell neuron precursors (GCNPs) after stimulation with Shh for 3h.	10	9.81E-4
TBK1.DF_DN	286	Genes down-regulated in epithelial lung cancer cell lines upon over-expression of an oncogenic form of KRAS [Gene ID=3845] gene and knockdown of TBK1 [Gene ID=29110] gene by RNAi.	13	1.07E-3
MTOR_UP.V1_DN	182	Genes down-regulated by everolimus [PubChem = 6442177] in prostate tissue.	10	1.52E-3
PGF_UP.V1_UP	190	Genes up-regulated in HUVEC cells (endothelium) by treatment with PGF [Gene ID=5281].	10	1.94E-3
CAMP_UP.V1_UP	200	Genes up-regulated in primary thyrocyte cultures in response to cAMP signaling pathway activation by thyrotropin (TSH).	10	2.66E-3

Table S36. Top ten most significantly enriched genesets of oncogenic collection (C6) for the top 500 genes of miRNA-gene pairs with most significant corrected p-values in IDC setting TBN.

Geneset Name	# Genes in Geneset	Description	# Genes in Overlap	FDR q-value
CAMP_UP.V1_DN	199	Genes down-regulated in primary thyrocyte cultures in response to cAMP signaling pathway activation by thyrotropin (TSH).	12	9.01E-4
CAMP_UP.V1_UP	200	Genes up-regulated in primary thyrocyte cultures in response to cAMP signaling pathway activation by thyrotropin (TSH).	11	1.87E-3
GCNP_SHH_UP_EARLY.V1_DN	167	Genes down-regulated in granule cell neuron precursors (GCNPs) after stimulation with Shh for 3h.	10	1.87E-3
STK33_UP	286	Genes up-regulated in NOMO-1 and SKM-1 cells (AML) after knockdown of STK33 [Gene ID=65975] by RNAi.	13	1.87E-3
ATF2_UP.V1_DN	185	Genes down-regulated in myometrial cells over-expressing ATF2 [Gene ID=1386] gene.	10	2.79E-3
PGF_UP.V1_UP	190	Genes up-regulated in HUVEC cells (endothelium) by treatment with PGF [Gene ID=5281].	10	2.9E-3
ERBB2_UP.V1_DN	197	Genes down-regulated in MCF-7 cells (breast cancer) positive for ESR1 [Gene ID=2099] and engineered to express ligand-activatable ERBB2 [Gene ID=2064].	10	3.02E-3
CSR_EARLY_UP.V1_DN	126	Genes down-regulated in early serum response of CRL 2091 cells (foreskin fibroblasts).	8	3.02E-3
TBK1.DF_UP	287	Genes up-regulated in epithelial lung cancer cell lines upon over-expression of an oncogenic form of KRAS [Gene ID=3845] gene and knockdown of TBK1 [Gene ID=29110] gene by RNAi.	12	3.57E-3
DCA_UP.V1_DN	185	Genes down-regulated in A549 lung carcinoma and M059K glioblastoma cells treated with dichloroacetate [PubChem=6597].	9	6.12E-3

Table S37. Top ten most significantly enriched genesets of oncogenic collection (C6) for the top 500 genes of miRNA-gene pairs with most significant corrected p-values in IDC setting ALLT.

Geneset Name	# Genes in Geneset	Description	# Genes in Overlap	FDR q-value
ERBB2_UP.V1_UP	190	Genes up-regulated in MCF-7 cells (breast cancer) positive for ESR1 [Gene ID=2099] and engineered to express ligand-activatable ERBB2 [Gene ID=2064].	13	8.41E-5
MTOR_UP.N4.V1_DN	184	Genes down-regulated in CEM-C1 cells (T-CLL) in comparison of control vs rapamycin (sirolimus) [PubChem=6610346], an mTOR pathway inhibitor.	12	1.78E-4
EIF4E_DN	100	Genes down-regulated in HMEC cells (primary mammary epithelium) upon over-expression of EIF4E [Gene ID=1977] gene.	9	1.78E-4
MTOR_UP.N4.V1_UP	196	Genes up-regulated in CEM-C1 cells (T-CLL) in comparison of control vs rapamycin (sirolimus) [PubChem=6610346], an mTOR pathway inhibitor.	12	1.85E-4
RAF_UP.V1_DN	193	Genes down-regulated in MCF-7 cells (breast cancer) positive for ESR1 [Gene ID=2099] MCF-7 cells (breast cancer) stably over-expressing constitutively active RAF1 [Gene ID=5894] gene.	11	7.29E-4
GCNP_SHH_UP_EARLY.V1_DN	167	Genes down-regulated in granule cell neuron precursors (GCNPs) after stimulation with Shh for 3h.	10	9.48E-4
STK33_SKM_UP	279	Genes up-regulated in SKM-1 cells (AML) after knockdown of STK33 [Gene ID=65975] by RNAi.	12	3.09E-3
SIRNA_EIF4GI_UP	95	Genes up-regulated in MCF10A cells vs knockdown of EIF4G1 [Gene ID=1981] gene by RNAi.	7	3.09E-3
TBK1.DF_UP	287	Genes up-regulated in epithelial lung cancer cell lines upon over-expression of an oncogenic form of KRAS [Gene ID=3845] gene and knockdown of TBK1 [Gene ID=29110] gene by RNAi.	12	3.45E-3
PDGF_ERK_DN.V1_UP	145	Genes up-regulated in SH-SY5Y cells (neuroblastoma) in response to PDGF [Gene ID=] stimulation after pre-treatment with the ERK inhibitors U0126 and PD98059 [PubChem=3006531, 4713].	8	6.12E-3

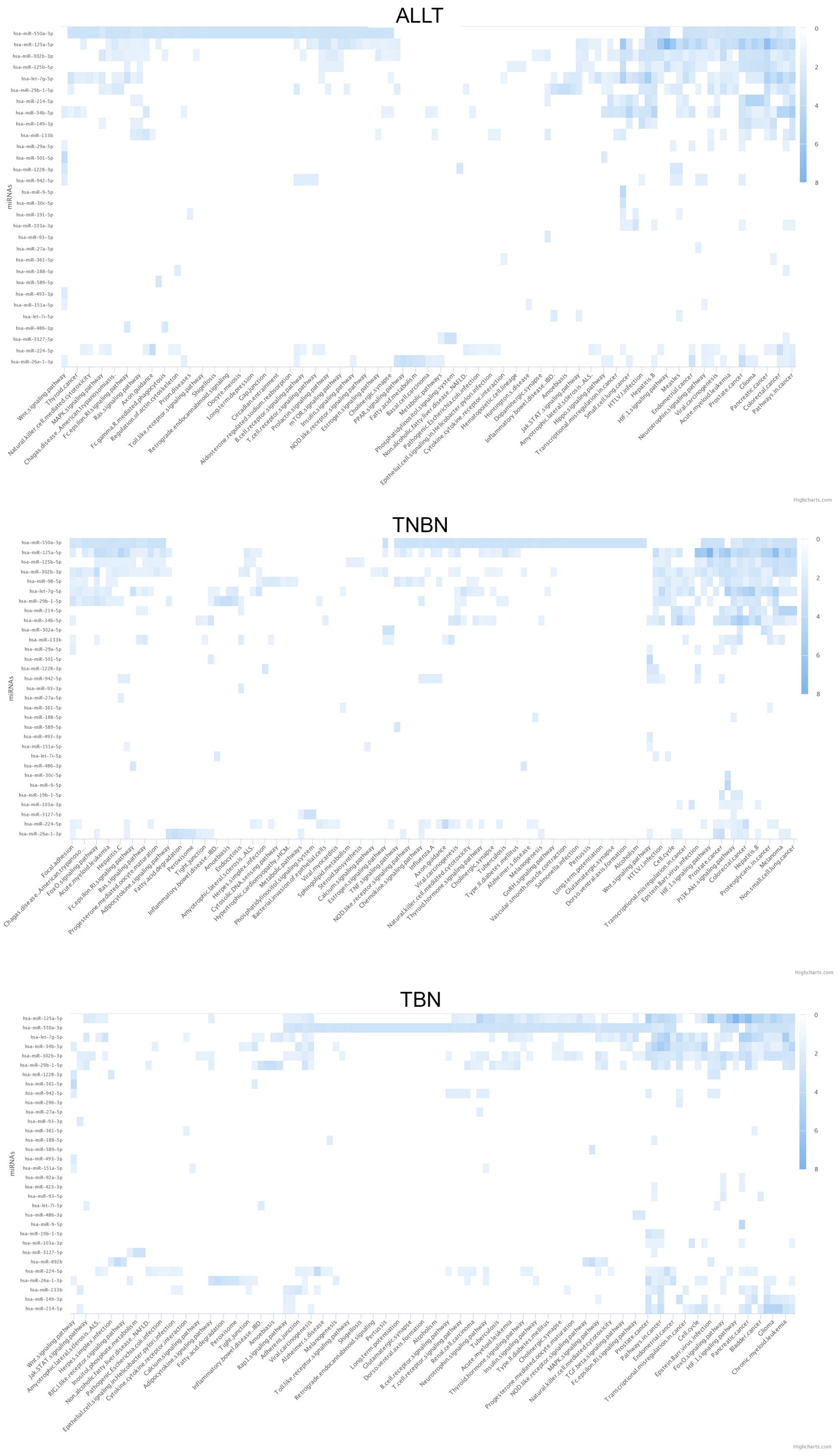

Figure S7. Heat maps as generated by miRPathDB for KEGG database using strong experimental evidence for the top 100 miRNAs with most significant corrected p-values for disease Brain lower grade glioma (LGG) for settings ALLT, TNBN, and TBN. On the y axis, the subset of miRNAs where any pathway was found to be significant are shown, on the x axis the significantly enriched pathways are shown. The intensity of the color represents -log(p-value).

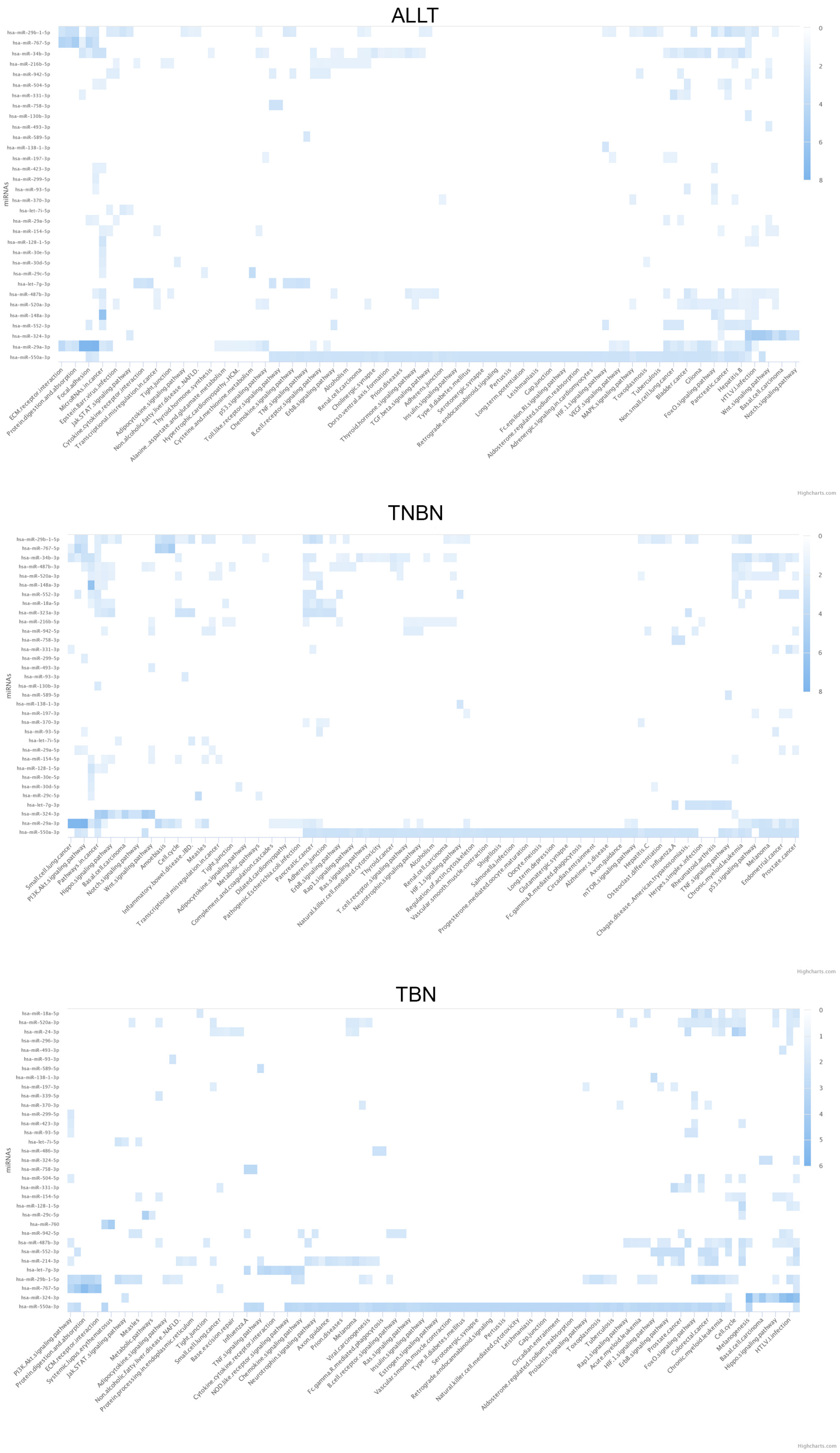

Figure S8. Heat maps as generated by miRPathDB for KEGG database using strong experimental evidence for the top 100 miRNAs with most significant corrected p-values for disease Kidney chromophobe carcinoma (KICH) for settings ALLT, TNBN, and TBN. On the y axis, the subset of miRNAs where any pathway was found to be significant are shown, on the x axis the significantly enriched pathways are shown. The intensity of the color represents -log(p-value).

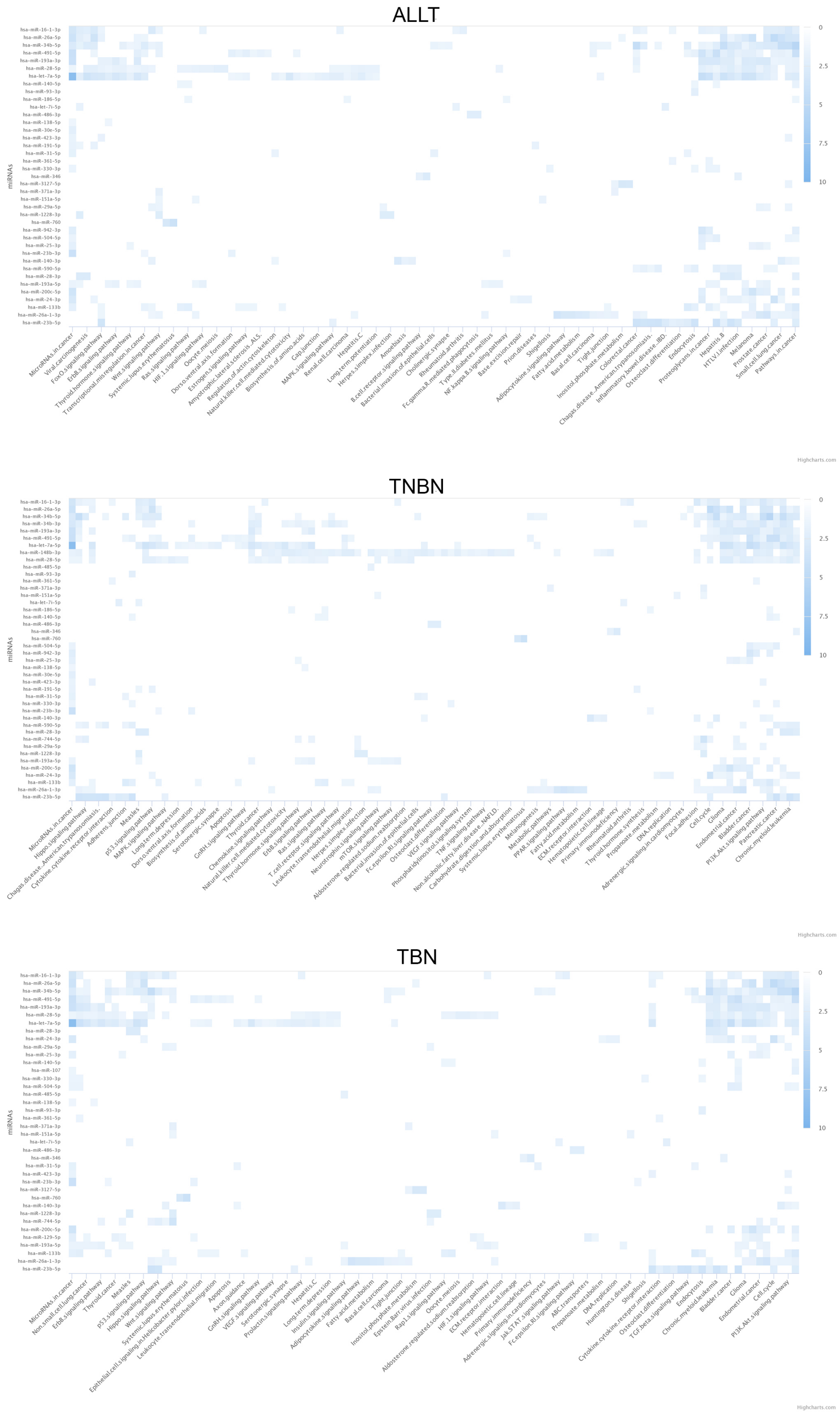

Figure S9. Heat maps as generated by miRPathDB for KEGG database using strong experimental evidence for the top 100 miRNAs with most significant corrected p-values for disease Liver hepatocellular carcinoma (LIHC) for settings ALLT, TNBN, and TBN. On the y axis, the subset of miRNAs where any pathway was found to be significant are shown, on the x axis the significantly enriched pathways are shown. The intensity of the color represents -log(p-value).

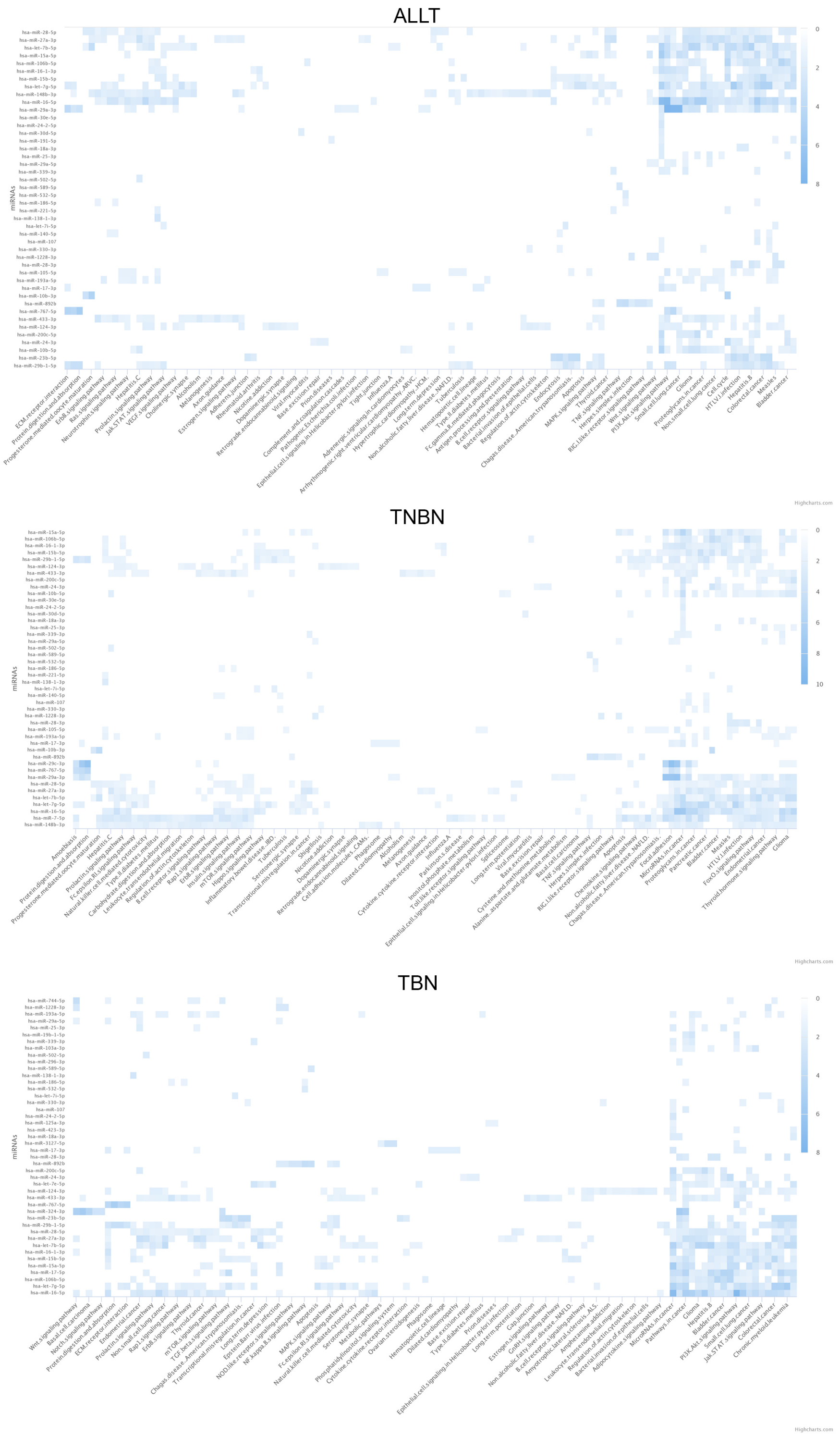

Figure S10. Heat maps as generated by miRPathDB for KEGG database using strong experimental evidence for the top 100 miRNAs with most significant corrected p-values for disease Kidney renal cell carcinoma (KIRC) for settings ALLT, TNBN, and TBN. On the y axis, the subset of miRNAs where any pathway was found to be significant are shown, on the x axis the significantly enriched pathways are shown. The intensity of the color represents -log(p-value).

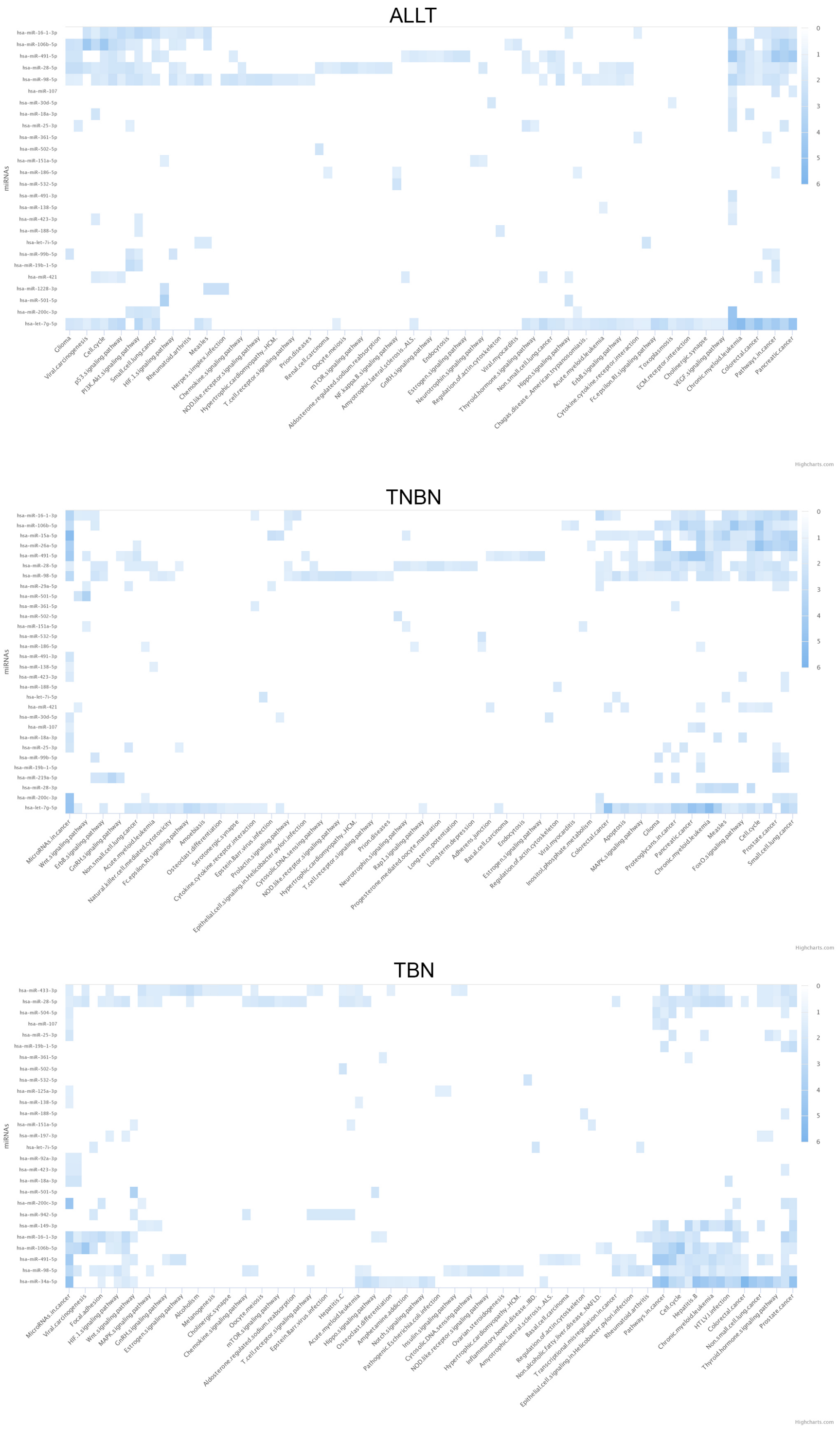

Figure S11. Heat maps as generated by miRPathDB for KEGG database using strong experimental evidence for the top 100 miRNAs with most significant corrected p-values for disease Invasive Lobular Carcinoma (ILC) for settings ALLT, TNBN, and TBN. On the y axis, the subset of miRNAs where any pathway was found to be significant are shown, on the x axis the significantly enriched pathways are shown. The intensity of the color represents -log(p-value).

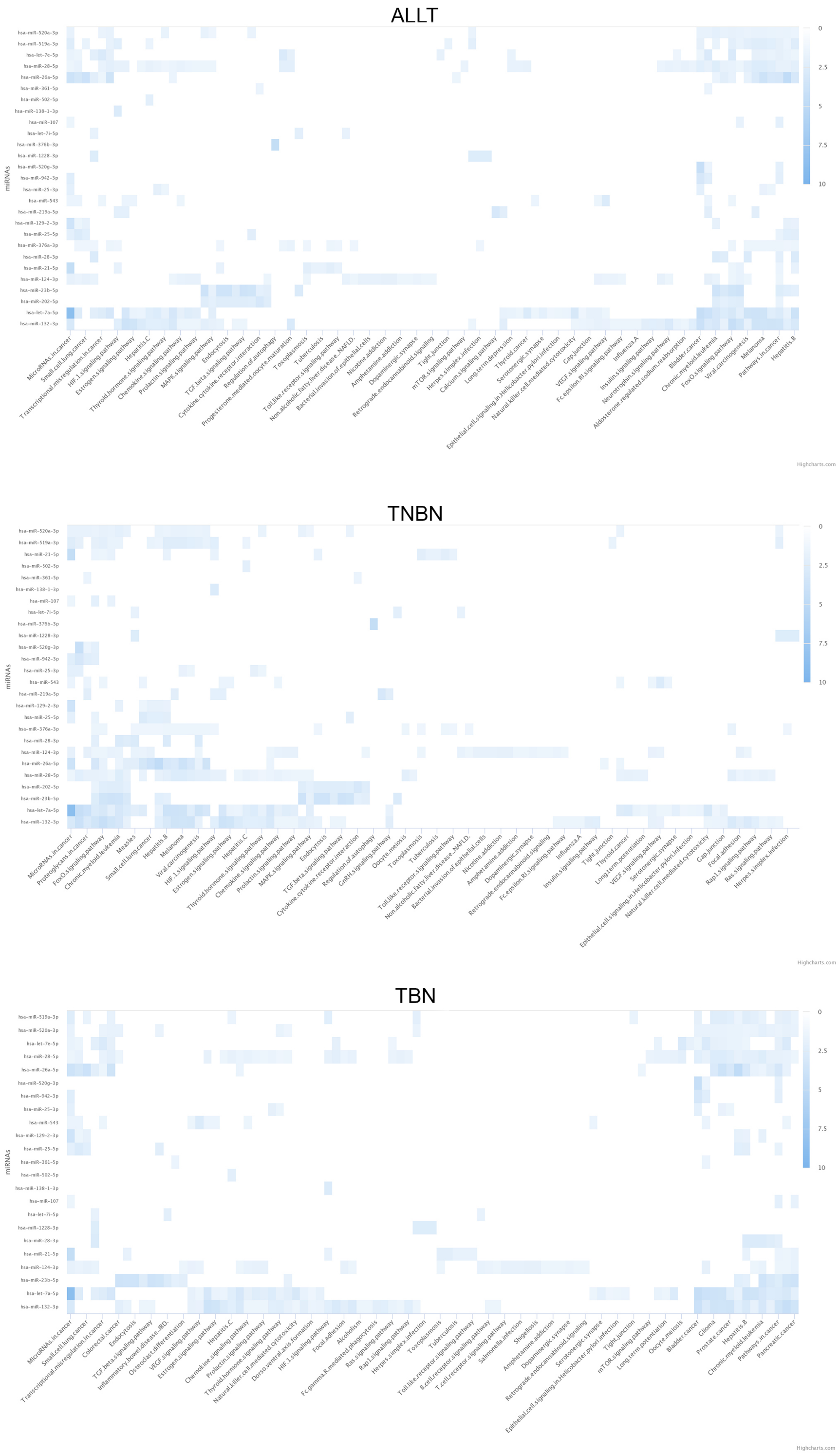

Figure S12. Heat maps as generated by miRPathDB for KEGG database using strong experimental evidence for the top 100 miRNAs with most significant corrected p-values for disease Invasive Ductal Carcinoma (IDC) for settings ALLT, TNBN, and TBN. On the y axis, the subset of miRNAs where any pathway was found to be significant are shown, on the x axis the significantly enriched pathways are shown. The intensity of the color represents -log(p-value).

Table S38. Intermediate number of miRNA-gene pairs after different filtering steps and number final nested models shown for settings ALLT, TNBN, TBN for Parkinson disease.

	# of miRNA-gene pairs after expression filter			# of miRNA-gene pairs after alternative splicing filter			# of miRNA-gene models after RMSE filter		
	ALLT	TNBN	TBN	ALLT	TNBN	TBN	ALLT	TNBN	TBN
PPMI	766,142	275,813	165,510	111,3101	479,312	258,411	138,441	47,919	26,932

Table S39. Number of samples, miRNAs, and miRNA-gene pairs after all filtering steps shown for settings ALLT, TNBN, TBN for Parkinson disease.

	# of samples	# of miRNAs	# of miRNA-gene pairs after all filters		
			ALLT	TNBN	TBN
PPMI	1,613	100	254,646	90,710	44,201

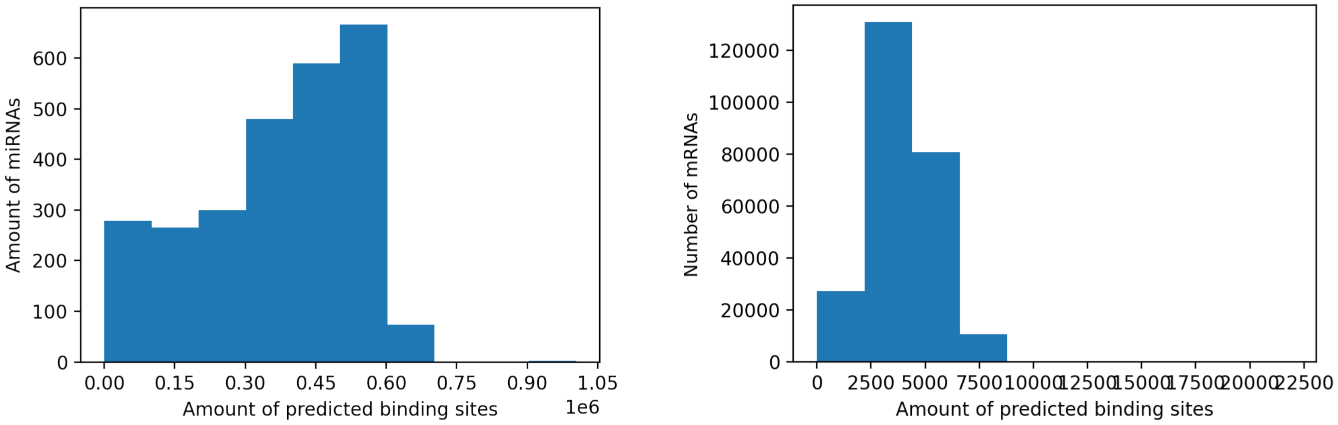

Figure S13. Distribution of predicted TarPmiR target sites before any filtering
